# Synthesis and Electrochemistry of New Furylpyrazolino[60]fullerene Derivatives by Efficient Microwave Radiation

**DOI:** 10.3390/molecules24244435

**Published:** 2019-12-04

**Authors:** Hamad M. Al-Matar, Mohammad H. BinSabt, Mona A. Shalaby

**Affiliations:** Chemistry Department, Faculty of Science, University of Kuwait, P.O. Box 5969, Safat 13060, Kuwait; mohammad.binsabt@ku.edu.kw (M.H.B.); mona_shalby2003@yahoo.com (M.A.S.)

**Keywords:** 1,3-dipolar cycloaddition, [60]fullerenes, furylpyrazoline, photophysical, electrochemical

## Abstract

Efficient one-pot synthesis of new series of furylpyrazolino[60]fullerene derivatives was prepared by [3 + 2] cycloaddition reaction mediated with (diacetoxyiodo)benzene (PhI(OAc)_2_) as an oxidant in *o*-dichlorobenzene (ODCB) under microwave irradiation. Different techniques have been used to confirm the structural identity including FT-IR, fast atom bombardment (FAB)-mass, NMR, and single-crystal X-ray diffraction, in addition to investigating the photophysical properties and the electrochemical properties for the new compounds using UV-Vis spectra, fluorescence spectra, cyclic voltammetry, and square wave voltammetry. Three of these pyrazolino[60]fullerene compounds showed better electron affinity than the parent C_60_ in the ground state.

## 1. Introduction

Since the first discovery of the well-known class of spherical molecules [1], fullerenes have attracted worldwide attention due to their unique structure and paved the way in many research areas [2,3,4,5]. The research interest in studying such unique molecules is due to their fascinating physical and chemical properties such as the capability of forming endohedral and exohedral derivatives, electrical properties, electron-accepting nature (which facilitates their use in preparation of photovoltaic cells), mechanical strength, and negligible biotoxicity [6]. [60]-Fullerene (C_60_) is one of the most widely studied fullerenes due to their perfect symmetry and availability [7]. Buckminsterfullerene (C_60_) is highly symmetrical (icosahedral *I_h_* with all 60 carbon atoms chemically equivalent) and is relatively reactive owing to their geometric constraints [8,9,10]. For all these characteristics, C_60_ possess a privileged status in the family of fullerenes. However, the limited solubility of C_60_ in some polar organic solvents and absolute insolubility in water, where it displays a high degree of aggregation, has hindered their use in many applications. This problem can be overcome by applying chemical modifications for C_60_. Functionalization of fullerenes using proper organic addend tunes their unique properties [11,12,13,14]. From a chemical point of view, the search for modified new fullerenes with unconventional properties for practical applications is still highly desirable [15]. C_60_-based molecules have a wide range of biomedical applications including radiotracers [16], magnetic resonance imaging (MRI) contrast agents for metallo-fullerenols [17], drug delivery systems [18], and photodynamic therapy (PDT) for the treatment of multiple diseases, mainly cancer [19,20]. On the other hand, 1,3-dipolar cycloaddition of nitrile imines to C_60_ have been widely used to graft the pyrazoline ring to the sphere of fullerene through different methods in moderate yields [21]. Although most of fullerene-based compounds decreases the electron-acceptor properties compared to the pristine C_60_ due to the double bond saturation in the C_60_ sphere [22], the electron affinity of pyrazolino[60]fullerenes have been improved [23,24] due to the inductive effect of the pyrazoline ring. Furthermore, recent studies on organic photovoltaic cells indicate that cells built with pyrazoline–C_60_ derivatives have a higher efficiency than those constructed with [6,6]-phenylC_61_-butyric acid methylester (PCBM) [25,26,27], which acts as a charge-separation module for the construction of photoactive and tunable devices suggesting that these compounds are good candidates to be studied [28,29].

Recently, these compounds have been prepared in one-step reaction mediated with PhI(OAc)_2_ as an oxidant [30,31]. Due to the excellent photophysical properties of 5-arylfurfural derivatives, these will be used here to functionalize C_60_ [32,33,34]. Microwave irradiation is a very useful tool in cycloaddition reactions [21,35] as well as in fullerene chemistry [36,37,38], where the target compounds are formed in reasonably good yields within a few minutes. The electrochemical properties of these compounds are investigated to show that the acceptor character is better or the same as C_60_. Herein, we report the synthesis, photophysical properties, and electrochemistry of novel series of furylpyrazolino[60]fullerene derivatives bearing different aryl groups.

## 2. Results

### 2.1. Synthesis

**Scheme 1:**
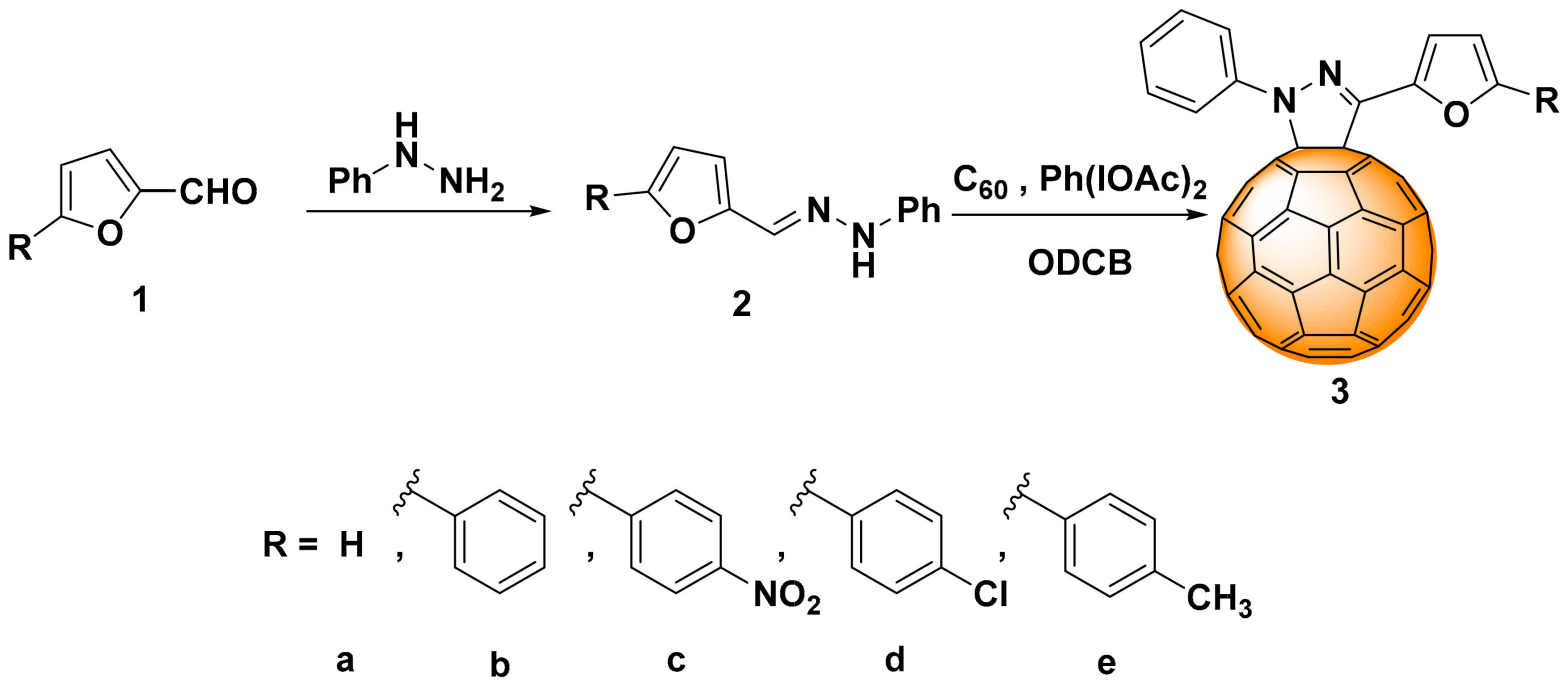
Synthesis of furylpyrazolino[60]fullerene derivatives **3a**–**e.**

**Scheme 2:**
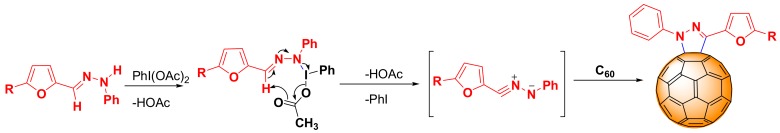
Possible mechanism for the formation of furylpyrazolino[60]fullerene derivatives **3a**–**e** using PhI(OAc)_2_ as an oxidant.

Scheme 1 illustrates the main strategy used to synthesize the target compounds **3a**–**e** based upon the [3 + 2] cycloaddition of C_60_ with nitrile imine derivatives generated in a one-pot protocol from the corresponding furylhydrazones **2a**–**e** using PhI(OAc)_2_ as an oxidant. The precursor compounds 5-arylfuran-2-carbaldehydes **1a**–**e** were synthesized according to the literature method [39,40]. The substituted furylhydrazones **2a**–**e** obtained from the corresponding aldehydes **1a**–**e** using the methodology previously reported [32]. A mixture of C_60_ (100 mg), substituted furylhydrazones **2a**–**e**, and PhI(OAc)_2_ was stirred in 10 mL *o*-dichlorobenzene (ODCB) for 60 min at 60 °C to afford the desired target compounds: furylpyrazolino[60]fullerene derivatives **3a**–**e**. Furthermore, we examined the same reactions under microwave irradiation (200 W), where the reaction was carried out at 60 °C for 30 min to afford compounds **3a**–**e**. Table 1 shows that microwave irradiation method (Method B) is better than conventional heating method (Method A) in both yield and time consumed. Reducing reaction time is considered an advantage as it reduces the production of undesired byproducts and prevents polcycloaddition. At first, the nitrile imine intermediate was generated due to the reaction between PhI(OAc)_2_ and substituted furylhydrazone. Then, the resulting intermediates undergo 1,3-dipolar cycloaddition reaction with C_60_ to form furylpyrazolino[60]fullerene derivatives **3a**–**e** as shown in Scheme 2 [30,31]. These cycloadduct products **3a**–**e** using microwave irradiation were purified using column chromatography (silica gel, toluene) and isolated using high-performance liquid chromatography (HPLC) (Buckyprep, toluene) affording good isolated yields (25–34%). The structural identification of the isolated furylpyrazolino[60]fullerene derivatives **3a**–**e** was confirmed using a variety of characterization techniques including: FT-IR, FAB-mass, NMR, UV-Visible, and single-crystal X-ray diffraction (see the experimental section and the electronic Supplementary Materials).

**Figure 1:**
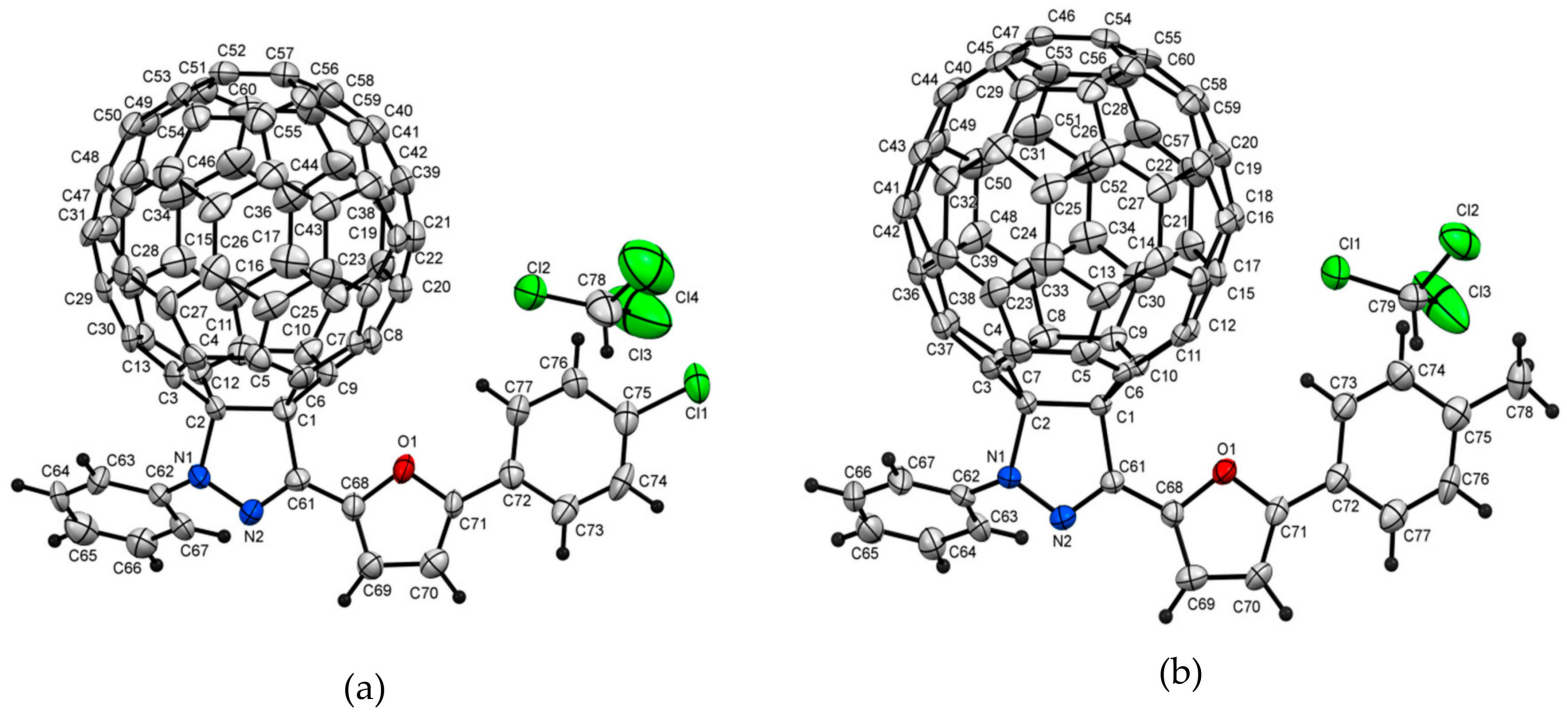
ORTEP diagrams of the crystal structures: (**a**) **3d** and (**b**) **3e**.

The molecular ion peaks for all target compounds **3a**–**e** were observed in the mass spectra using FAB at *m*/*z* values of the expected M^+^ fragment. The ^1^H NMR spectra of the pure cycloadduct products **3a**–**e** show all the expected signals of the organic addend. Moreover, ^13^C NMR and two-dimensional (HSQC and HMBC) NMR spectroscopy used to determine the structure identity. All of the prepared compounds **3a**–**e** showed a moderate or weak absorption band at around 430 nm in their UV-Vis spectra, which is characteristic of the 1,2-dihydrofullerene [41]. The products **3d** and **3e** were further confirmed by single-crystal X-ray crystallography as shown in Figure 1.

### 2.2. Crystal Structure

**Figure 2:**
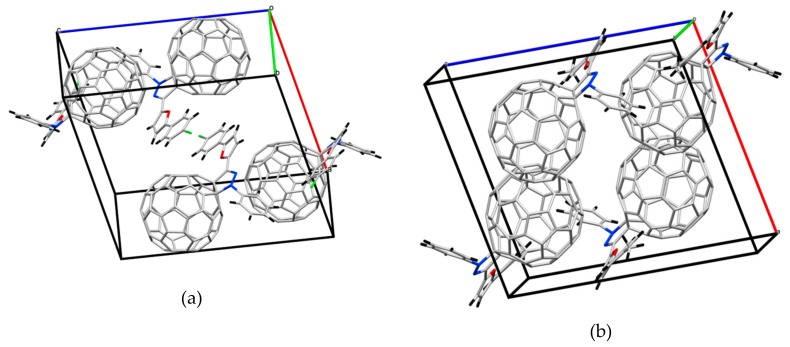
The unit cell contents and the molecular packing arrangements of (**a**) **3d** and (**b**) **3e**.

Crystals of compounds **3d** and **3e** were grown by a diffusion technique using the chloroform/toluene solvent system. Blocks-like crystals of these compounds were obtained within one week and are found to be stable for the X-ray diffraction studies. The crystal structure of both compounds is monoclinic with the *P*2_1_/*c* space group at 150 K. Although both X-ray crystal structures show almost identical structural morphology, deviations in the packing arrangement are observed (Figure 2).

The structures of both molecules are consistent with those expected from their synthetic procedure and confirm well with other characterization techniques. The bottom end of the fullerene molecule is functionalized with a pyrazoline moiety that attached to a phenyl group in one side and a substituted aryl furan on the other side where these aryl groups are 4-chlorophenyl and 4-methylphenyl groups, respectively, in the case of **3d** and **3e** systems. While the aryl substituted furan moiety is almost coplanar with the pyrazoline ring, the phenyl ring is oriented almost perpendicular with respect to the pyrazoline unit in both crystals. The torsion angle between the plane of phenyl ring and the pyrazoline moiety is 105^o^ and 104^o^ for **3d** and **3e**, respectively.

**Figure 3:**
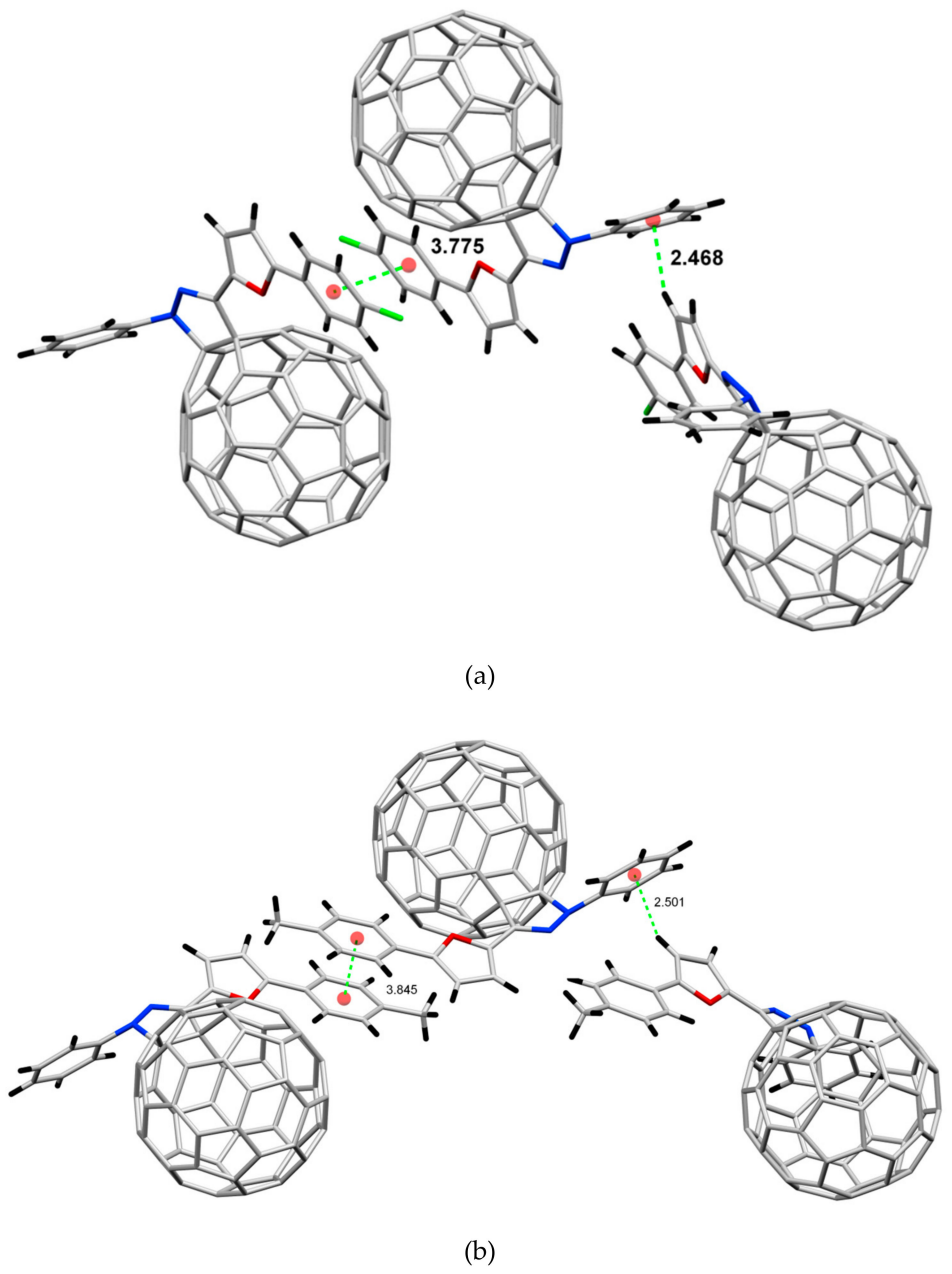
Demonstration of π…π and C-H… π interactions exhibited by (**a**) **3d** and (**b**) **3e** toward its neighboring molecules in crystal network. Color code: Blue-nitrogen; gray-carbon; red-oxygen; green-chlorine and black-hydrogen.

The packing of both compounds in their crystal is efficiently achieved by utilizing intermolecular nonbonding interactions with adjacent fullerenes. The 4-chlorophenyl or 4-methylphenyl moieties of adjacent fullerenes are capable of engaging efficient π–π interactions in their crystals as demonstrated in Figure 3. The aryl–aryl distance in **3d** and **3e** crystals are 3.775 Å and 3.845 Å, respectively, and these are well within the range to enable strong π–π interactions. The perpendicularly oriented phenyl ring in this structure is also able to engage π…H-C type interaction with a third fullerene molecule in the neighborhood (Figure 3). These C-H… π interaction distances are in the range of 2.468 Å and 2.501 Å for **3d** and **3e** crystals, respectively. Both interactions are sufficient to make these crystals stable enough to form efficient supramolecular networks [42].

### 2.3. UV-Vis Spectra

**Figure 4:**
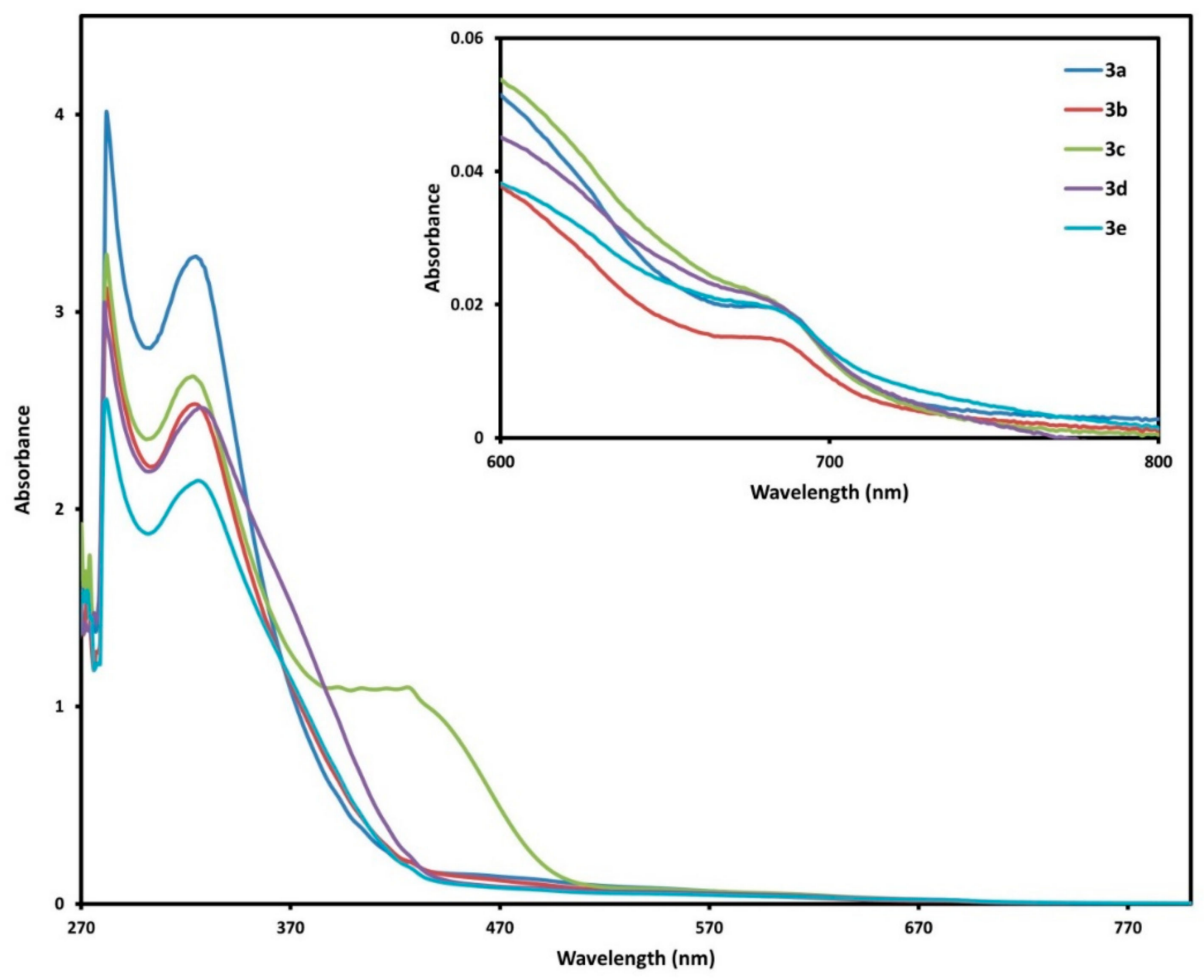
UV-Vis spectra of **3a**–**e** in toluene solutions at room temperature (c = 5 × 10^−5^ mol/L). Inset shows the expansion from 600 to 800 nm.

**Figure 5:**
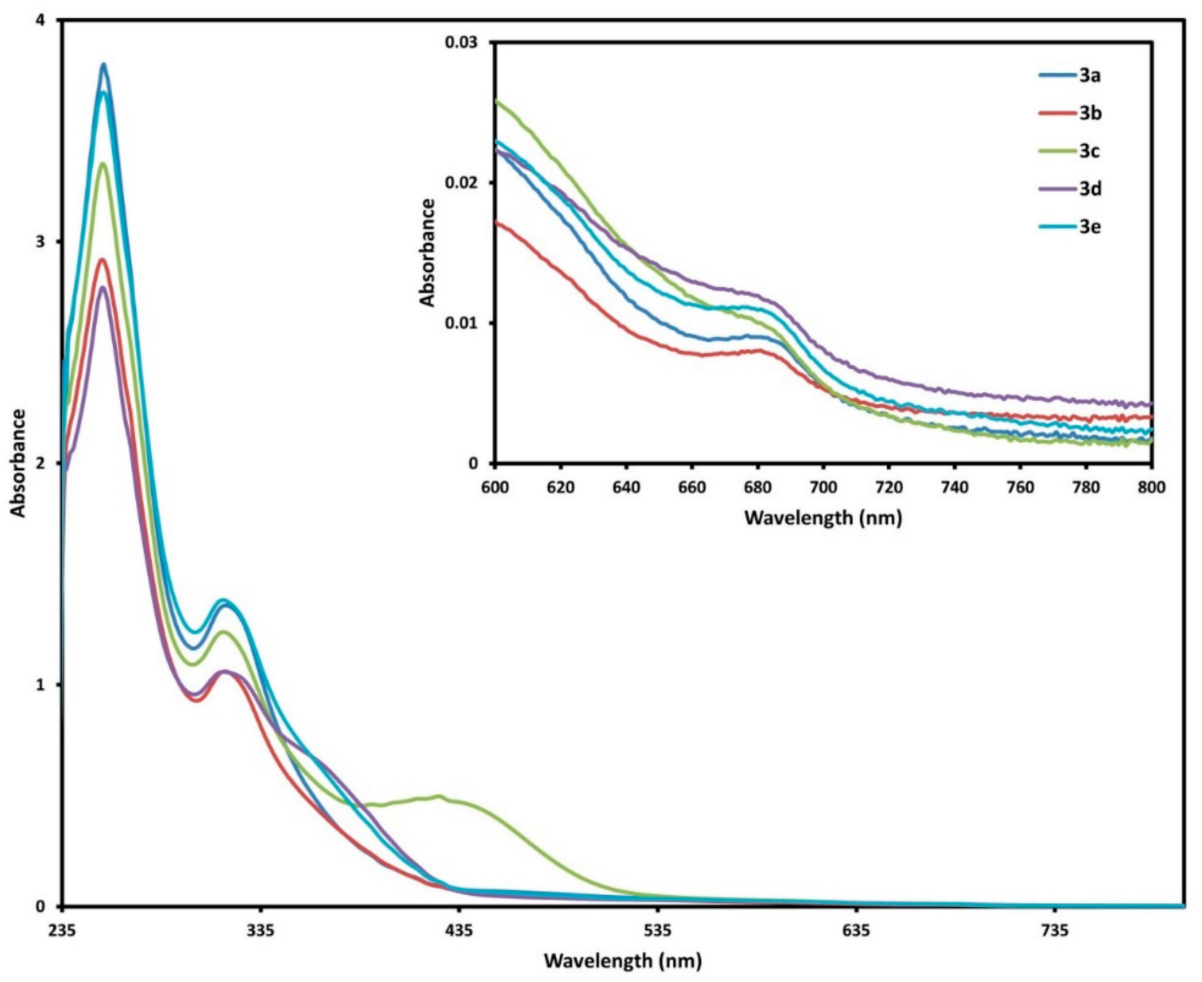
UV-Vis spectra of **3a**–**e** in chloroform solutions at room temperature (c = 2.5 × 10^−5^ mol/L). Inset shows the expansion from 600 to 800 nm.

The UV-Vis spectra of **3a**–**e** in nonpolar solvent toluene and the more polar solvent chloroform are shown in Figure 4 and Figure 5, respectively. All compounds exhibit two main absorption band peaks at 282 and 325 nm in toluene while in chloroform they exhibit peaks at 250 and 317 nm where in this region the absorption bands of fullerene and the functionalized group may be overlapped. In addition, a weak peak is observed at 688 nm in both solvents (toluene and chloroform) for all compounds as in most C_60_-cycloadducts which was observed at 700 nm [43]. The broad absorption band in the region 450–750 nm corresponds to an S_0_→S_1_ transition. Compound **3c** displays peak at around 426 nm in toluene and chloroform which is a characteristic peak for this compound than the other synthesized compounds. This peak is attributed to the strong ground state electronic interaction between the C_60_ moieties and the substituent group through pyrazoline group than the other substituent groups. Furthermore, a shoulder was observed for compounds **3d** and **3e** in toluene at 379 and 390 nm, respectively, while in chloroform observed at 374 and 370 nm, respectively. These data revealed that compound **3c,** which has the *p*-nitrophenyl group attached at position 5 of furyl group, has the higher main wave length than the other derivatives.

### 2.4. Fluorescence Spectra

**Figure 6:**
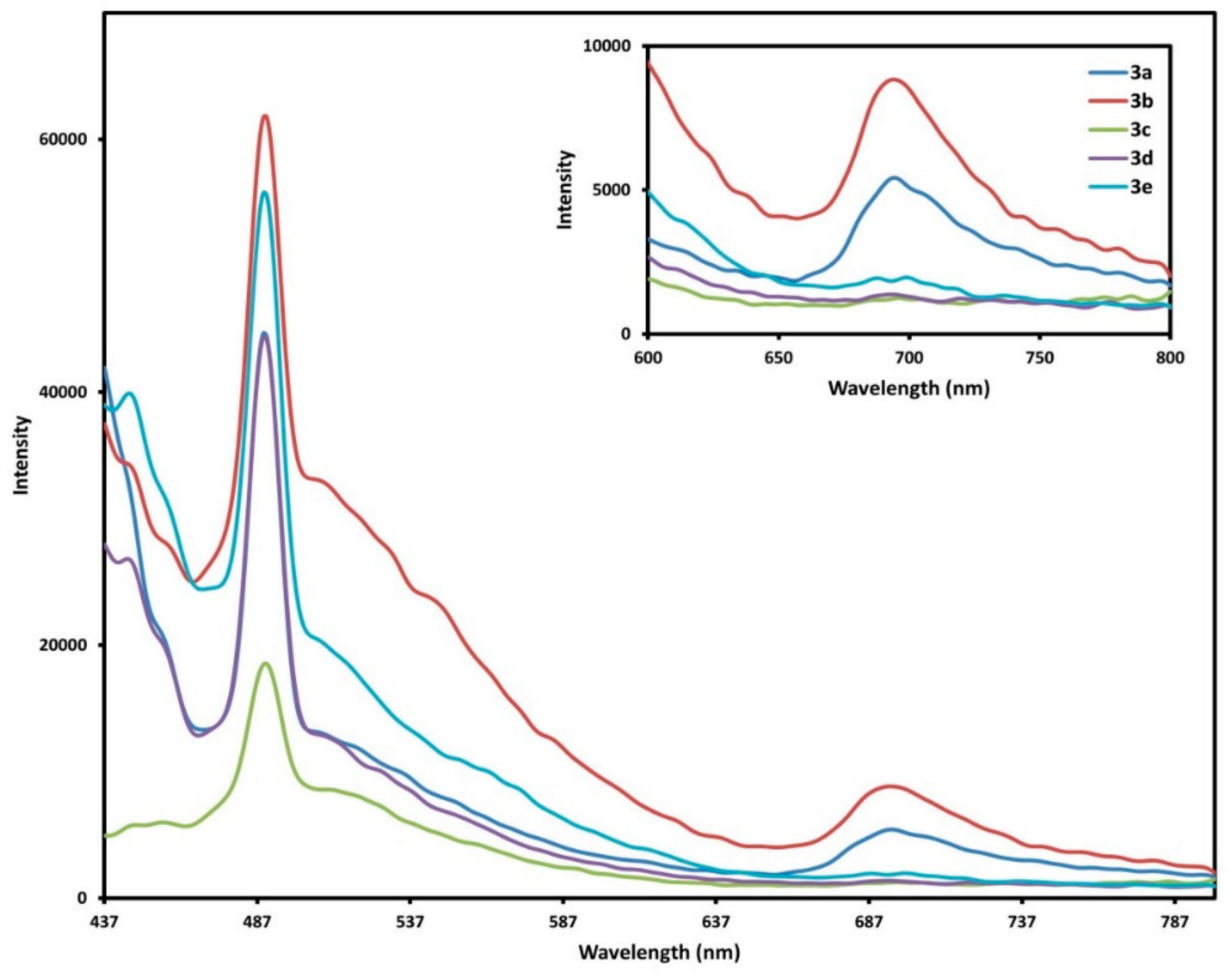
Fluorescence spectra of **3a**–**e** in toluene at room temperature (concentrations are kept at 0.05 mM; *λ*_ex_ = 427 nm). Inset shows the expansion from 600 to 800 nm.

**Figure 7:**
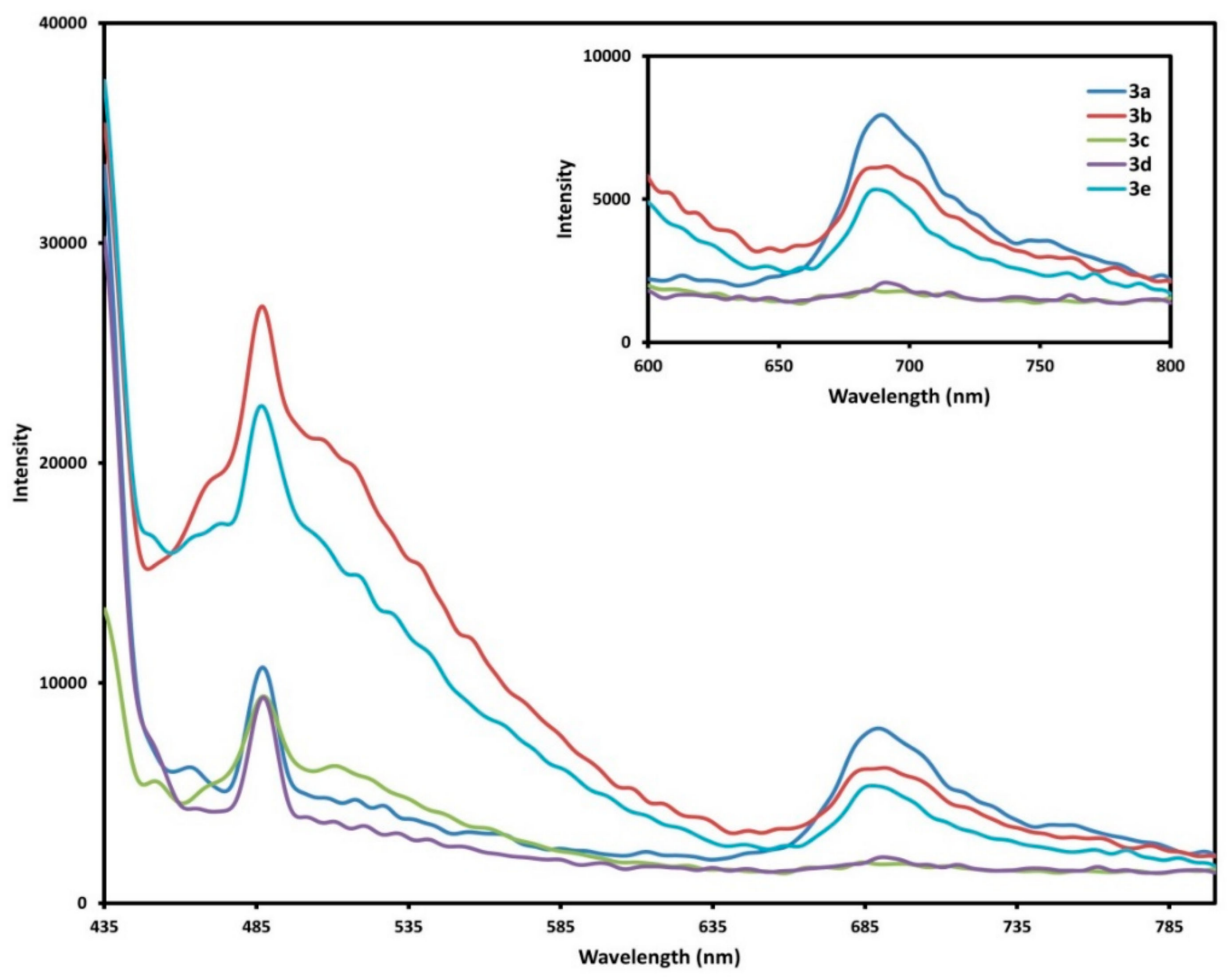
Fluorescence spectra of **3a**–**e** in chloroform at room temperature (concentrations are kept at 0.05 mM; *λ*_ex_ = 427 nm). Inset shows the expansion from 600 to 800 nm.

The fluorescence spectra of **3a**–**e** were measured in both toluene and chloroform upon excitation wavelength at 427 nm in which the excited state (S_1_) of C_60_ moiety (^1^C_60_^*^) was generated at room temperature (Figure 6 and Figure 7). Both solvents show similar behavior for all compounds. Compounds **3a**–**e** exhibited two fluorescence peaks at 490 and 691 nm. The observed broad fluorescence peak at 490 nm may be due to the group attached to C_60_ moiety and the second observed one at 691 nm is due to fullerene moiety which clearly found in compounds **3a**, **3b,** and **3e**. In case of compounds **3c** and **3d**, the fluorescence peak is totally quenched even by using toluene as a solvent. This quenching may be due to the charge separation process which is not common in nonpolar solvent [44]. As the electronic deficiency of the substituent attached to furyl group increased, the fluorescence intensity of product **3** became weaker in toluene (i.e., **3b** >**3a** > **3e** > **3d** and **3c**).

### 2.5. Electrochemistry

**Figure 8:**
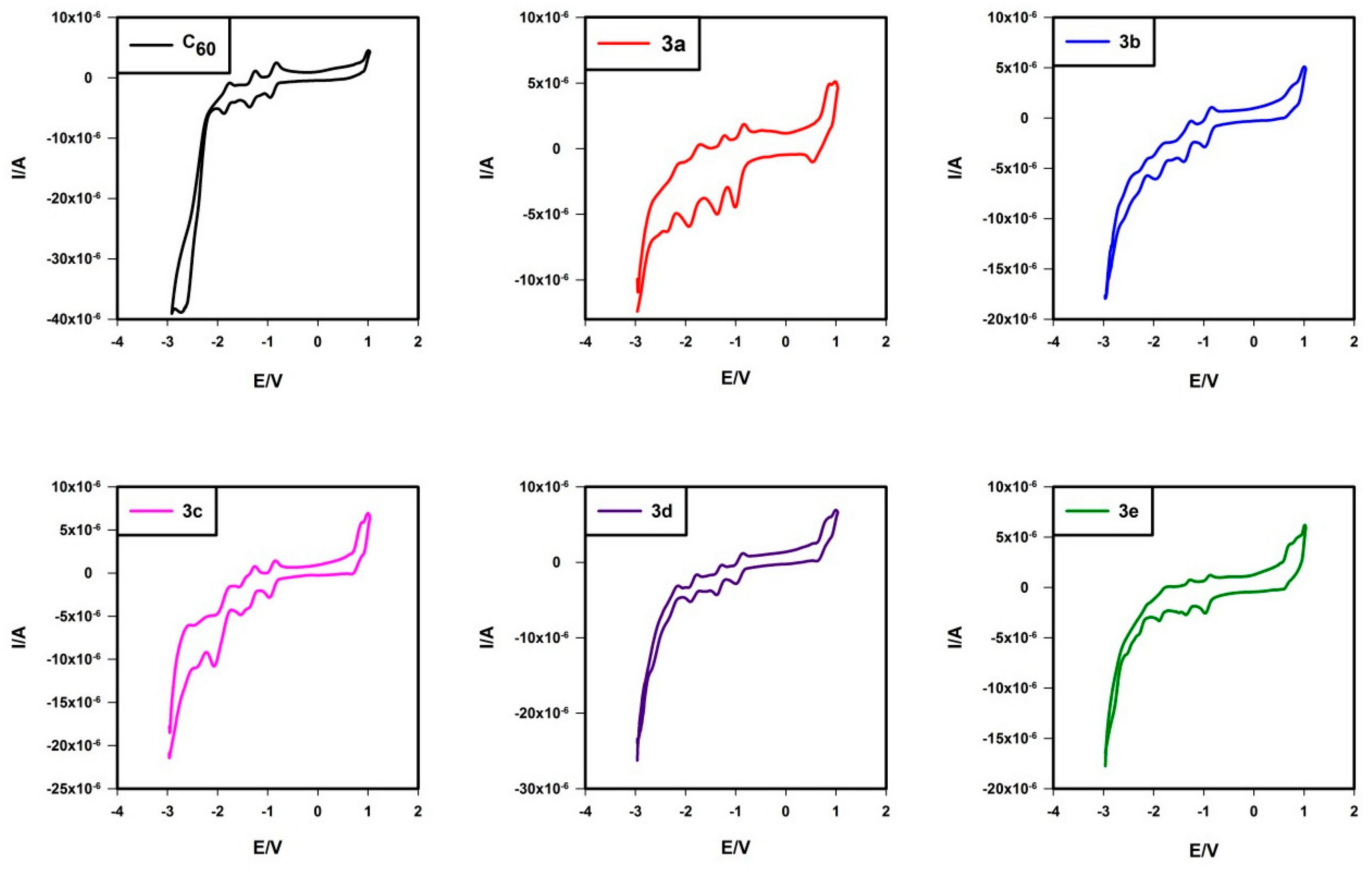
Cyclic voltammogram of C_60_ and furylpyrazolino[60]fullerene derivatives **3a**–**e** in toluene/CH_3_CN (4:1) and 0.1 M tetrabutylammonium perchlorate (TBAP); V vs. Ag/AgCl; scan rate was 50 mVs^−1^.

The electrochemical properties of C_60_ and furylpyrazolino[60]fullerene derivatives **3a**–**e** with 0.1 M tetrabutylammonium perchlorate (TBAP) as supporting electrolyte in toluene/acetonitrile solution were studied using cyclic voltammetry (CV) and square-wave voltammetry (SWV) at room temperature. The resulting CV and SWV data are summarized in Figure 8 and Table 2 along with the data for C_60_. All compounds **3a**–**e** showed the four one-electron reduction peaks, which assigned to reduction of the C_60_ cage. For compounds **3a**–**e**, reversible reduction peaks between the main potential peaks of the C_60_ moiety were observed that are probably due to the overlapping of two signals, one corresponding to the C_60_ cage and the other to the organic addend. It is noteworthy that the first reduction potential value of compounds **3a**, **3b**, and **3d** shows an anodic shift relative to C_60_ and the remaining furylpyrazolino[60]fullerene derivatives show shift at slightly more negative values (29–35 mV) comparing with that of C_60_. The first reversible reduction potential was studied in order to investigate the influence of the substituent attached to the carbon atom (C-3) of the pyrazolino system when benzene ring is present in (N-1) side of the same ring system. From Table 2, the $E_{red}^{1}\;$ values shift to more negative in the order of **3c** > **3e** > **3b** > **3d** > **3a**. Consequently, compounds **3a**, **3b**, and **3d** have higher accepting properties (electron affinity) than the parent C_60_ while the other compounds have a slight decreasing of the accepting properties. On the oxidation side, the first oxidation $E_{ox}^{1}$ is due to the organic addend.

## 3. Materials and Methods 

### 3.1. General Methods

All cycloaddition reactions were performed in standard glassware under an inert atmosphere of argon. C_60_ was purchased from Sigma Aldrich (St. Louis, Missouri USA). All used solvents were purchased from Aldrich (Muskegon, MI, USA). Thin layer chromatography (TLC) was performed using Polygram SIL G/UV 254 TLC plates from Merc (Darmstadt, Germany), and visualization was performed under ultraviolet light at 254 and 350 nm. Column chromatography was performed using Merck silica gel 60 of mesh size 0.040−0.063 mm. Melting points were recorded on a Griffin melting point apparatus and are reported uncorrected. Mass analyses were done by electron impact (EI) and fast atom bombardment (FAB) on a Thermo DFS mass spectrometer. A high-resolution mass analysis (HRMS) was performed on a Xevo G2-S QTof mass spectrometer from Waters (USA). IR spectra were obtained with a Jasco 6300 FTIR (Japan). ^1^H-NMR (600 MHz) and ^13^C-NMR (150 MHz) spectra were recorded at 25 °C using Bruker DPX 400 or 600 superconducting NMR spectrometer (Bruker, Karlsruhe, Germany). UV-Vis studies were performed on a Varian Cary 5 spectrometer from Agilent (California, USA). Fluorescence measurements were carried out with Horiba Jobin Yvon-Fluoromax-4 equipped with a Time Correlated Single Photon Counting (TCSPC) module (Japan). Single-crystal data collections (Rigaku, Tokyo, Japan) were performed on a Rigaku R-Axis Rapid diffractometer using filtered Mo Kα radiation. The diffraction data were collected at a temperature of −123 °C (Oxford Cryosystems). Microwave experiments were carried out using a CEM Discover Labmate microwave apparatus (300 W with CHEMDRIVER software; Matthews, NC, USA). Reactions were conducted under microwave irradiation in heavy-walled Pyrex tubes fitted with PCS caps (closed vessel under pressure). A Waters HPLC instrument equipped from Waters (USA) with a pump (Waters 1525EF), a UV/visible detector (Waters 2487). The chromatographic separation was carried out in a cosmosil buckyprep HPLC column (10 × 250 mm). HPLC separations were performed by injecting 1000 μL with an isocratic toluene mobile phase at a flow rate of 2 mL/min. Cyclic voltammetry measurements were carried out on a GAMRY INSTRUMENTS (Warminster, PA 18974, USA) reference 3000 potentiostat using Ag/0.01 M AgCl (Model 6.0733.100, Metrohm), 0.1 M TBAP in ACN reference electrode, an auxiliary electrode consisting of a Pt sheet, and a MF-2012 glassy carbon electrode (3 mm) as a working electrode, directly immersed in the solution. Ferrocene (Fc) was added as an internal reference and all the potentials were referenced relative to the Fc/Fc^+^ couple. Scan rate: 50 mV s^−1^.

### 3.2. Synthesis

#### 3.2.1. General Synthesis Procedure of Substituted Hydrazones (2a–e) 

It was synthesized as reported in the literature [32].

#### 3.2.2. General Synthesis Procedure of Cycloadducts (3a–e)

**Method A**. A mixture of C_60_ (100 mg, 0.139 mmol), substituted hydrazones (**2a**–**e**) (0.139 mmol), and PhI(OAc)_2_ (0.139 mmol) was dissolved in 10 mL of ODCB and stirred at 60 °C for 1 h. The reaction progress was monitored by TLC with toluene as an eluent. At the end of the reaction, hexane was added, and the formed precipitate was filtered then purified on silica gel column using toluene as eluent to afford adducts (**3a**–**e**). The reaction products **3a**–**e** were separated by HPLC using toluene as an eluent.

**Method B**. In a similar process, the mixture was irradiated by microwave irradiation (200 W) for 30 min at 60 °C.

*1’-Phenyl-3’-(2-furyl)pyrazolino[4‘,5’:1,2]*[60]*fullerene* (**3a**). This compound was reported in the literature previously [45].

*1’-Phenyl-3’-(5-phenyl-2-furyl)pyrazolino[4‘,5’:1,2]*[60]*fullerene* (**3b**). Brown solid; FT-IR (KBr) ν (cm^−1^) 3434.6, 1725, 1665.2, 1596.7, 1489.7, 1443.4, 1259.2, 1170.5, 1129.1, 869.7, 802.2, 693.2, 527.4. ^1^H NMR (CDCl_3_) *δ* 6.9 (d, 1 H, *J* = 3.6 Hz), 7.24–7.29 (m, 2H), 7.34 (t, 2H, *J* = 7.8 Hz), 7.42 (d, 1H, *J* = 3.6 Hz), 7.50 (t, 2H, *J* = 6 Hz), 7.62 (d, 2H, *J* = 7.2 Hz), 7.98 (d, 2H, *J* = 7.2 Hz); ^13^C NMR (CDCl_3_) *δ* 155.6, 147.8, 147.6, 147.4, 146.9, 146.6, 146.5, 146.3, 146.2, 146.1, 146.0, 145.8, 145.7, 145.4,144.8, 144.5, 144.4, 143.3, 143.1, 143.0, 142.7, 142.6, 142.5, 142.4, 142.0, 140.2, 139.9, 136.5, 136.2, 130.2, 129.5, 129.0, 128.1, 125.7, 124.4, 124.1, 113.8, 107.7, 91.7, 80.1, 68.3, 38.9, 30.5, 29.9, 29.1, 23.9, 23.2, 14.28, 11.19. UV-Vis (Toluene) *λ*_max_ (nm) (log ε) 688 (2.44), 325 (4.70), 282 (4.79); UV-Vis (CHCl_3_) *λ*_max_(nm) (log *ε*) 681 (2.50), 320 (4.62), 255 (5.06). FAB-MS *m*/*z*: 981 (M+1), 721 (C_60_ + 1); HRMS (ESI-TOF) (*m*/*z*): (M + 1) calculated for C_77_H_12_N_2_O 981.1028 and found 981.1075.

*1’-Phenyl-3’-(5-(4-nitrophenyl)-2-furyl)pyrazolino[4’,5’:1,2]*[60]*fullerene* (**3c**). Dark brown solid; FT-IR (KBr) ν (cm^−1^) 3413.3, 1725.0, 1596.7, 1513.8, 1491.6, 1327.7, 1261.2, 1108.8, 851.4, 794.5, 750.1, 692.3, 527.4. ^1^H NMR (CS_2_/CDCl_3_) *δ* 7.12 (d, 1 H, *J* = 4.2 Hz), 7.28–7.30 (m, 1H), 7.44 (d, 1H, *J* = 4.2 Hz), 7.48 (t, 2H, *J* = 8.4 Hz), 7.71 (d, 2H, *J* = 9 Hz), 7.95 (d, 2H, *J* = 8.4 Hz), 8.18 (d, 2H, *J* = 9 Hz); ^13^C NMR (CS_2_/CDCl_3_) *δ* 152.7, 149.7, 147.7, 147.2, 146.6, 146.5, 146.4, 146.3, 146.1, 146.0, 145.8, 145.7, 145.6, 145.3, 145.2, 145.1, 144.9, 144.4, 144.3, 144.1, 143.2, 143.0, 142.9, 142.5, 142.4, 142.3, 142.2, 141.9, 140.1, 139.8, 136.4, 136.3, 135.4, 134.9, 129.4, 125.7, 124.4, 124.1, 124.0, 113.6, 111.3. UV-Vis (Toluene) *λ*_max_ (nm) (log *ε*) 687 (2.58), 426 (4.34), 323 (4.72), 282 (4.81); UV-Vis (CHCl_3_) *λ*_max_ (nm) (log ε) 683 (2.58), 320 (4.68), 255 (5.12). FAB-MS *m*/*z*: 1025 (M^+^), 720 (C_60_); HRMS (ESI-TOF) (*m*/*z*): (M + 1) calculated for C_77_H_12_N_3_O_3_ 1026.0879 and found 1026.0923.

*1’-Phenyl-3’-(5-(4-chlorophenyl)-2-furyl)pyrazolino[4’,5’:1,2]*[60]*fullerene* (**3d**). Black solid; FT-IR (KBr) ν (cm^−1^) 3134.7, 1719.2, 1588.1, 1508.0, 1475.2, 1350.8, 1305.5, 1244.8, 1210.1, 1108.8, 1090.5, 1041.3, 955.5, 863.9, 828.2, 789.7, 752.1, 691.3, 524.5. ^1^H NMR (CS_2_/CDCl_3_) *δ* 6.88 (d, 1 H, *J* = 3.6 Hz), 7.27 (m, 1H), 7.29 (d, 2H, *J* = 9 Hz), 7.40 (m, 1H), 7.49 (t, 2H, *J* = 7.8 Hz), 7.52 (d, 2H, *J* = 8.4 Hz), 7.95 (d, 2H, *J* = 7.8 Hz); ^13^C NMR (CS_2_/CDCl_3_) *δ* 154.1, 147.7, 147.6, 147.2, 146.5, 146.4, 146.3, 146.0, 145.9, 145.7, 145.5, 145.3, 145.2, 144.4, 144.3, 144.2, 143.1, 142.9, 142.8, 142.5, 142.4, 142.3, 142.2, 141.8, 140.0, 139.7, 136.4, 136.3, 133.8, 129.3, 129.0, 128.5, 128.3, 125.4, 125.1, 123.9, 113.6, 108.0. UV-Vis (Toluene) *λ*_max_ (nm) (log ε) 687 (2.58), 327 (4.70), 281 (4.78); UV-Vis (CHCl_3_) *λ*_max_ (nm) (log ε) 684 (2.66), 320 (4.62), 255 (5.04). FAB-MS *m*/*z*: 1015 (M^+^), 720 (C_60_); HRMS (ESI-TOF) (*m*/*z*): (M^+^) calculated for C_77_H_12_N_2_OCl 1015.0638 and found 1015.0645.

*1’-Phenyl-3’-(5-(4-methylphenyl)-2-furyl)pyrazolino[4’,5’:1,2]*[60]*fullerene* (**3e**). Dark brown solid; FT-IR (KBr) ν (cm^−1^) 3432.6, 1723.0, 1625.7, 1592.9, 1529.2, 1488.7, 1428.9, 1348.9, 1256.4, 1180.2, 1110.8, 1043.3, 865.8, 811.8, 784.8, 750.1, 691.3, 526.4. ^1^H NMR (CS_2_/CDCl_3_) *δ* 2.27 (s, 1H, CH_3_), 6.73 (d, 1 H, *J* = 3.6 Hz), 7.03 (d, 2H, *J* = 7.8 Hz), 7.14 (m, 1H), 7.28 (d, 1H, *J* = 3.6 Hz), 7.40 (m, 4H), 7.86 (d, 2H, *J* = 9 Hz); ^13^C NMR (CS_2_/CDCl_3_) *δ* 155.6, 147.6, 147.2, 147.0, 146.7, 146.5, 146.4, 146.3, 146.1, 146.0, 145.9, 145.8, 145.5, 145.3, 145.2, 144.7, 144.4, 144.2, 143.1, 142.9, 142.8, 142.5, 142.4, 142.3, 142.2, 141.8, 140.0, 139.7, 137.8, 136.4, 136.3, 135.7, 130.5, 129.5, 129.3, 129.0, 128.3, 127.6, 127.4, 125.3, 124.0, 123.9, 113.7, 106.9, 80.0, 91.4. UV-Vis (Toluene) *λ*_max_ (nm) (log *ε*) 691 (2.53), 426 (4.63), 282 (4.70); UV-Vis (CHCl_3_) *λ*_max_ (nm) (log ε) 681 (2.64), 320 (4.73), 256 (516). FAB-MS *m*/*z*: 995 (M+1), 721 (C_60_ + 1); HRMS (ESI-TOF) (*m*/*z*): (M + 1) calculated for C_78_H_15_N_2_O 995.1184 and found 995.1231.

## 4. Conclusions

We have synthesized a new series of furylpyrazolino[60]fullerene derivatives via [3 + 2] cycloaddition reaction mediated with PhI(OAc)_2_ as an oxidant under microwave irradiation. These compounds were investigated using the FT-IR, FAB-mass, NMR, UV-Vis, fluorescence spectra, electrochemical properties, and single-crystal X-ray diffraction. According to the first reduction potential, compounds **3a**, **3b**, and **3d** showed better electron affinity than the parent C_60_ in the ground state. Furthermore, compound **3c** has a higher main wave length than the other derivatives. While the fluorescence intensity for compounds **3d** and **3c** is totally quenched even on using toluene as a solvent which may be due to the charge separation process. The results are encouraging to continue studying the photophysical and the photochemical measurements for these compounds that are potentially useful for applications in material science.

## Figures and Tables

**Table 1:** Synthesis of furylpyrazolino[60]fullerene derivatives under conventional and microwave conditions.

Method A ^a^				Method B ^b^	
Entry	Product	Yield ^c^ (%)	Recovered C_60_ %	Yield ^c^ (%)	Recovered C_60_ %
2a	**3a**	15	44	20	30
2b	**3b**	15	50	25	35
2c	**3c**	19	40	30	30
2d	**3d**	20	42	34	33
2e	**3e**	19	43	25	34

^a^ Reaction of substituted furylhydrazone, C_60_, and PhI(OAc)_2_ in ODCB under conventional conditions (60 °C) for 1 h. ^b^ Reaction of substituted furylhydrazone, C_60_, and PhI(OAc)_2_ in ODCB under microwave irradiation for 30 min. ^c^ Isolated yields based on the reacted C_60_.

**Table 2:** Redox potentials of C_60_ and furylpyrazolino[60]fullerene derivatives **3a**–**e** determined by square-wave voltammetry (SWV) ^a.^

	$E_{red}^{1}$	$E_{red}^{2}$	$E_{red}^{3}$	$E_{red}^{4}$	$E_{ox}^{1}$
**3a**	−1.048	−1.457	−1.911	−2.374	0.807 *^b^*
**3b**	−1.055	−1.451	−1.993 (−2.106)	−2.416 (−2.581)	0.755 *^b^*
**3c**	−1.093	−1.410 (−1.616)	−1.885	−2.419	0.839 *^b^*
**3d**	−1.054	−1.432	−1.949	−2.336	0.788 *^b^*
**3e**	−1.087	−1.477	−1.961	−2.316	0.710 *^b^*
**C_60_**	−1.058	−1.458	−1.959	−2.472	

*^a^* Experimental conditions: V vs. Fc/Fc^+^; reference electrode is Ag/AgCl; working electrode is GCE; 0.1 M *n*Bu_4_NClO_4_; scan rate: 50 mVs^−1^; measured in toluene/CH_3_CN (4:1 *v*/*v*) at room temperature. *^b^* Measured by CV.

## Supplementary Materials

The following are available online: ^1^H and ^13^C-NMR spectra, HSQC, HMBC, HPLC chromatogram, and HRMS (ESI-TOF) for each compound. The crystallographic data for molecule **3d** and **3e** have been deposited with the Cambridge Crystallographic Data Centre as the supplementary publication number CCDC 1959808 and 1959811.
