# Supplementary material for: Synthesis and Electrochemistry of New Furylpyrazolino[60]fullerene Derivatives by Efficient Microwave Radiation
Source: Molecules. 2019 Dec 4;24(24):4435. doi: 10.3390/molecules24244435 (PMC6943683; doi:10.3390/molecules24244435)
Supplement: Supplementary file 1 [file molecules-24-04435-s001.pdf]

Supporting information for

Synthesis and electrochemistry of new furylpyrazolino[60]fullerene derivatives by efficient microwave radiation

Hamad M. Al-Matar*, Mohammad H. BinSabt and Mona A. Shalaby

Chemistry Department, Faculty of Science, University of Kuwait, P.O. Box 5969, Safat 13060, Kuwait

*Corresponding author. Tel.: +965 24987559; fax: +965 24816482.

E-mail address: h.almatar@ku.edu.kw (H.M. Al-Matar).

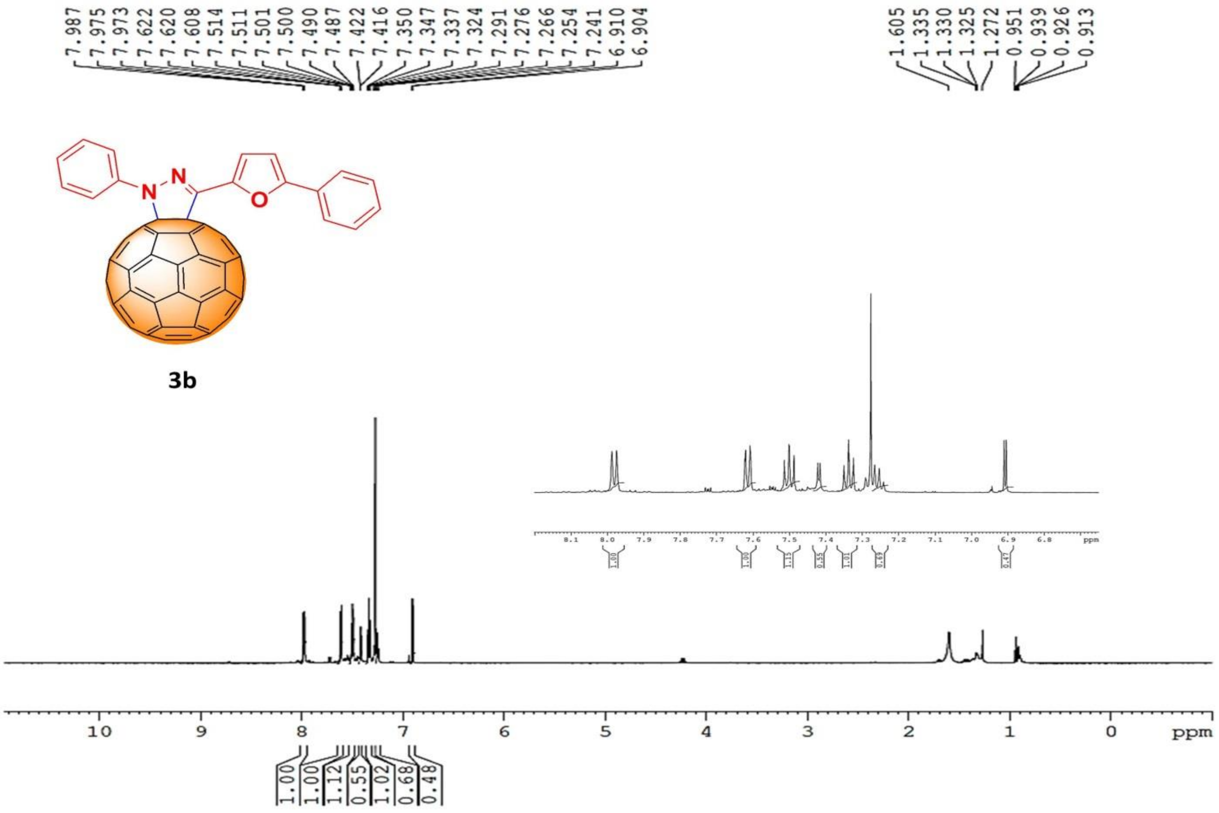

Figure 1. The ^{1}H NMR spectrum of compound **3b** (600.13 MHz, solvent $CDCl_3$)

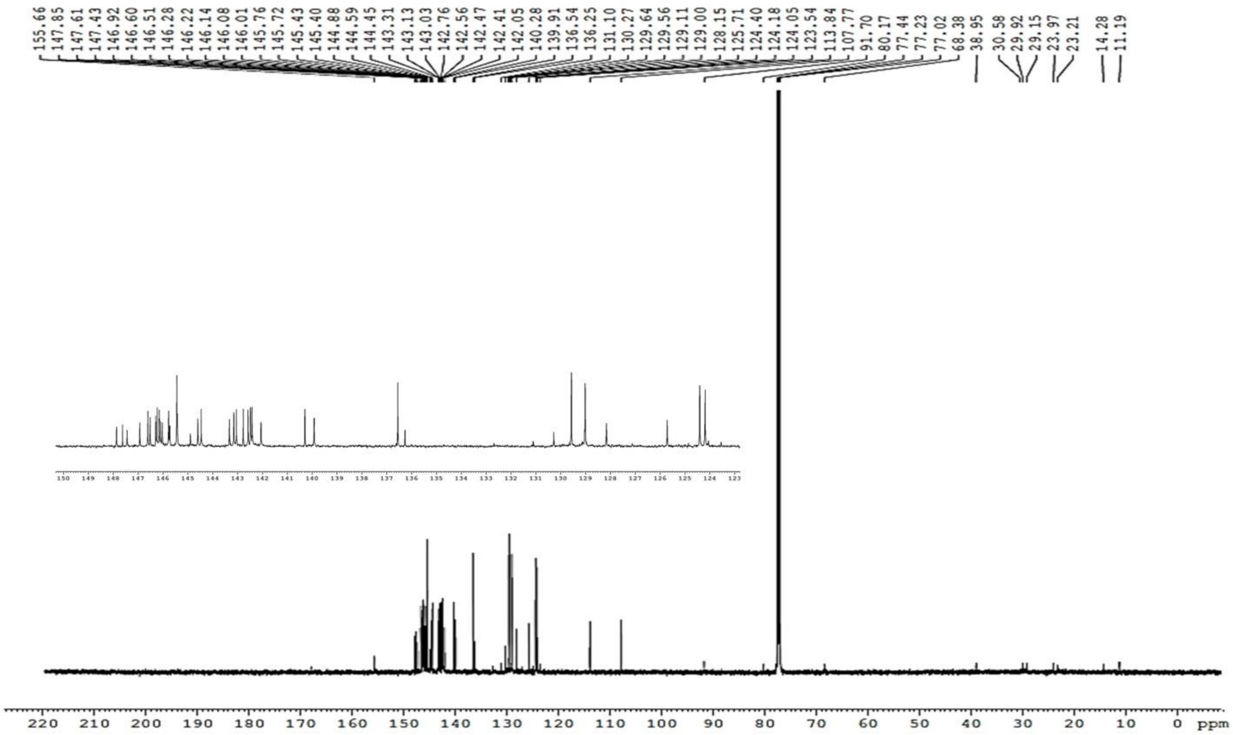

Figure 2. The ^{13}C NMR spectrum of compound **3b** (600.13 MHz, solvent $CDCl_3$)

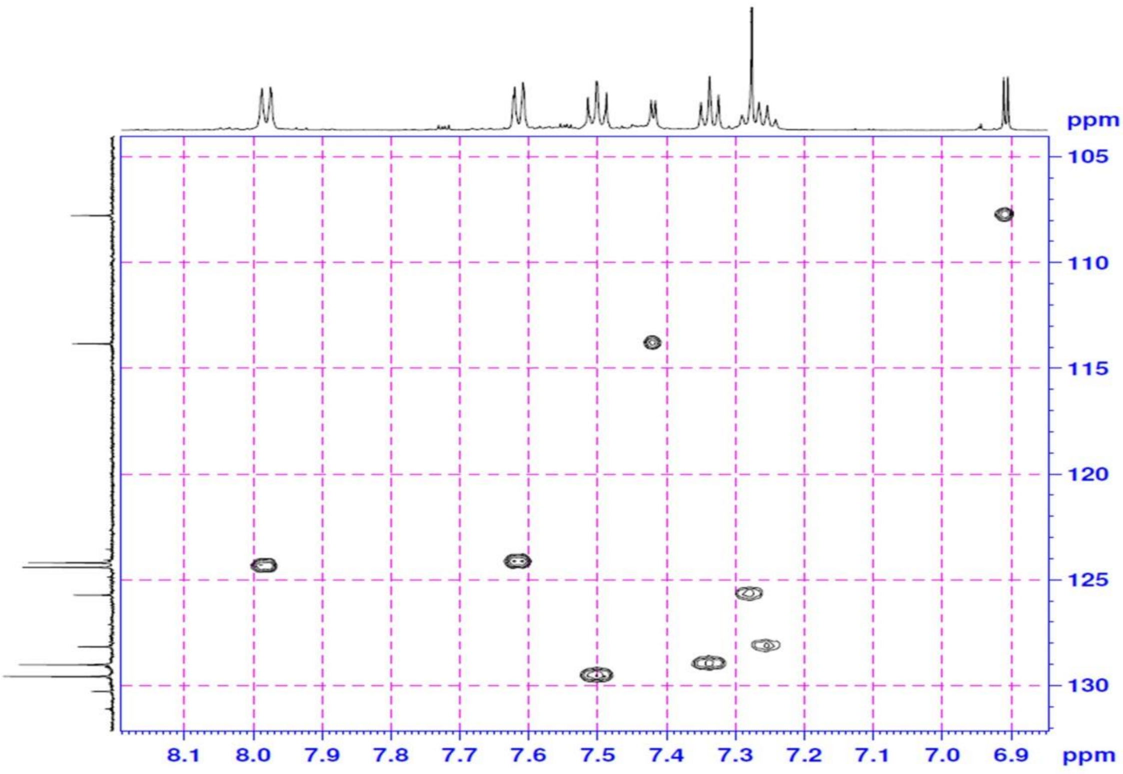

Figure 3. The HSQC spectrum of compound **3b** (600.13 MHz for ^{1}H and ^{13}C, solvent $CDCl_3$)

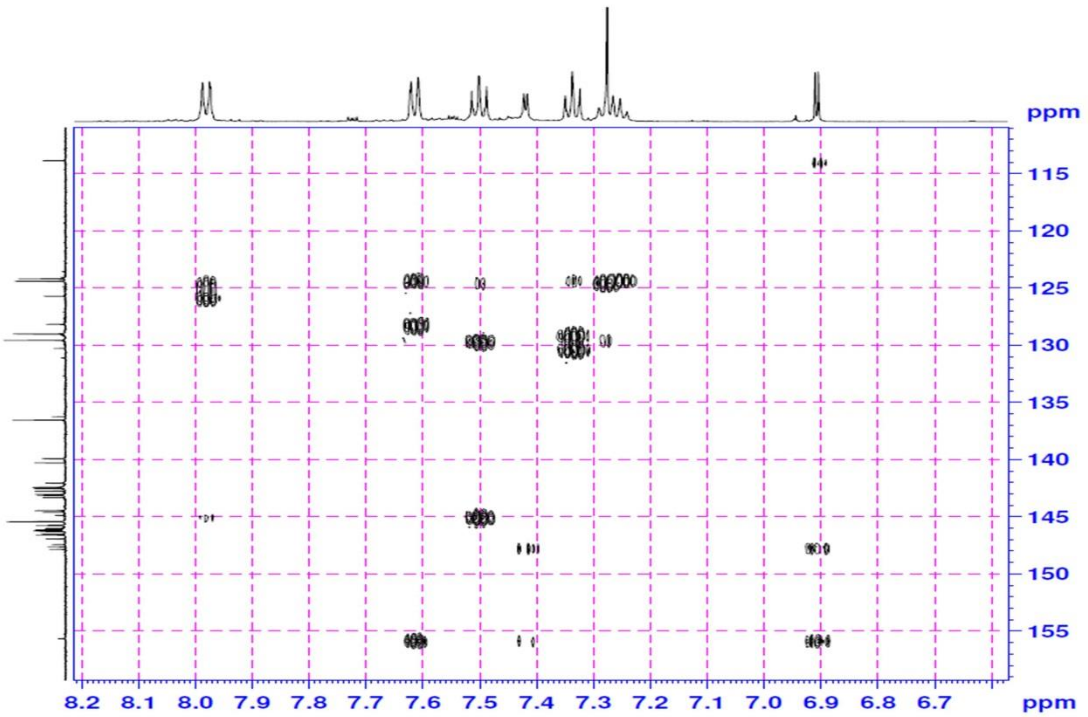

Figure 4. The HMBC spectrum of compound **3b** (600.13 MHz for ^{1}H and ^{13}C, solvent $CDCl_3$)

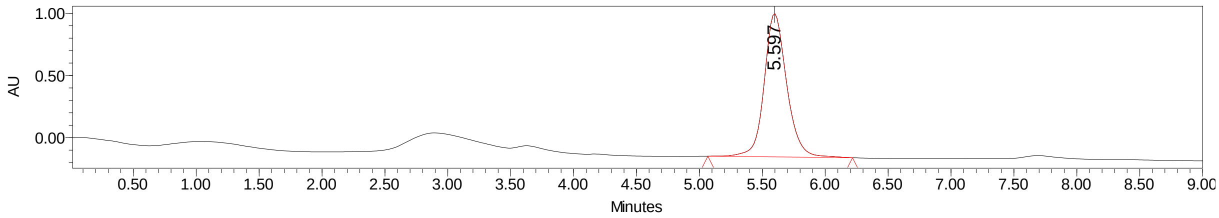

	Retention Time (min)	Area (V*sec)	% Area	Height (v)	% Height
1	5.597	14362860	100.00	1151675	100.00

Figure 5. HPLC chromatogram of compound **3b**

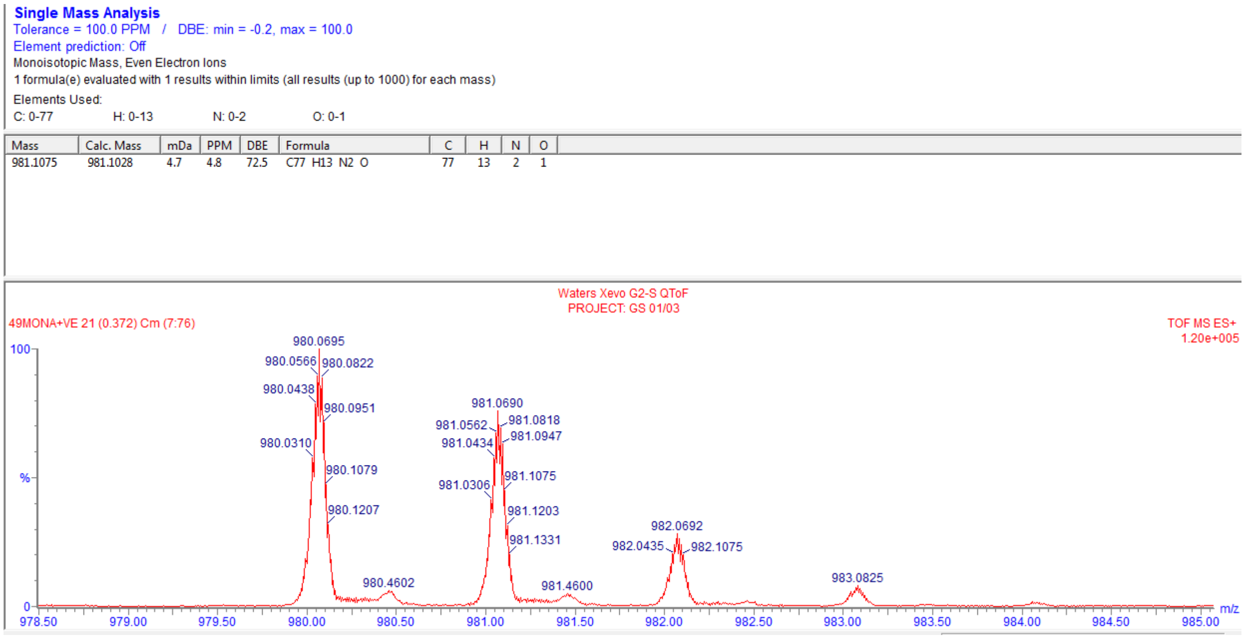

Figure 6. HRMS (ESI-TOF) of compound **3b**

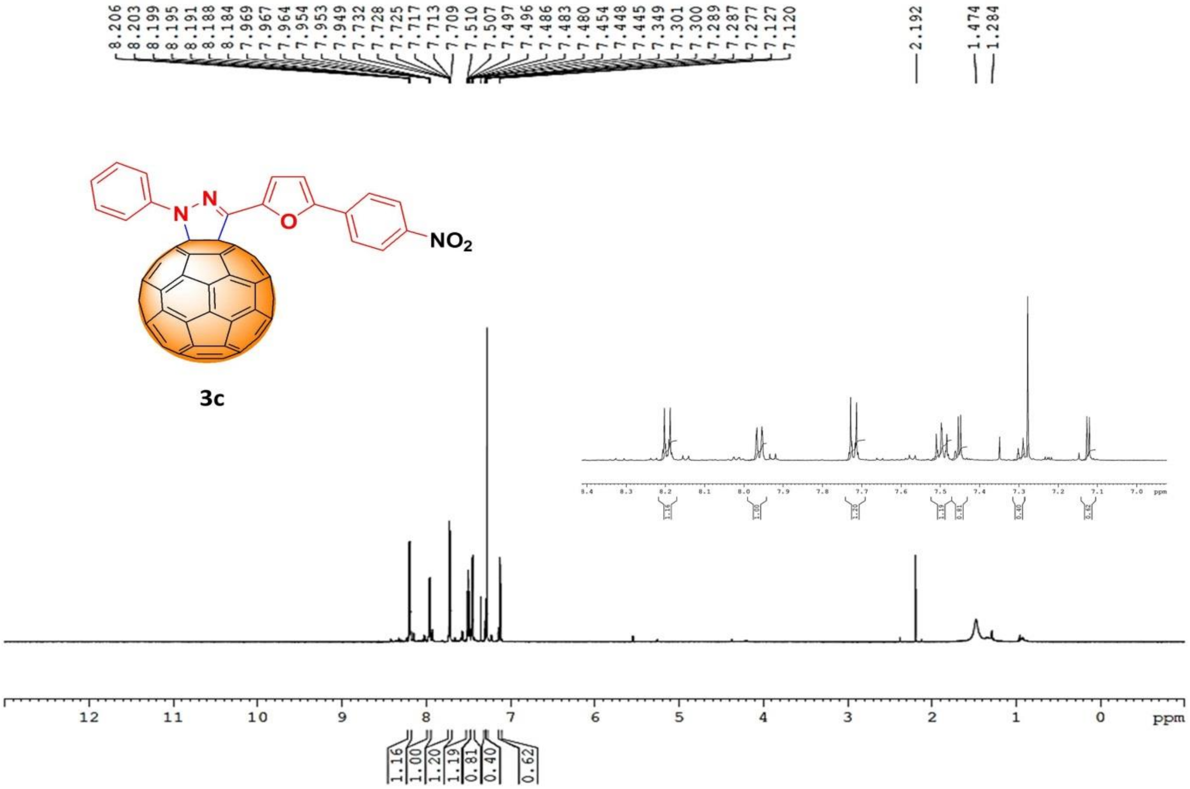

Figure 7. The ^1H NMR spectrum of compound **3c** (600.13 MHz, solvent $CDCl_3$)

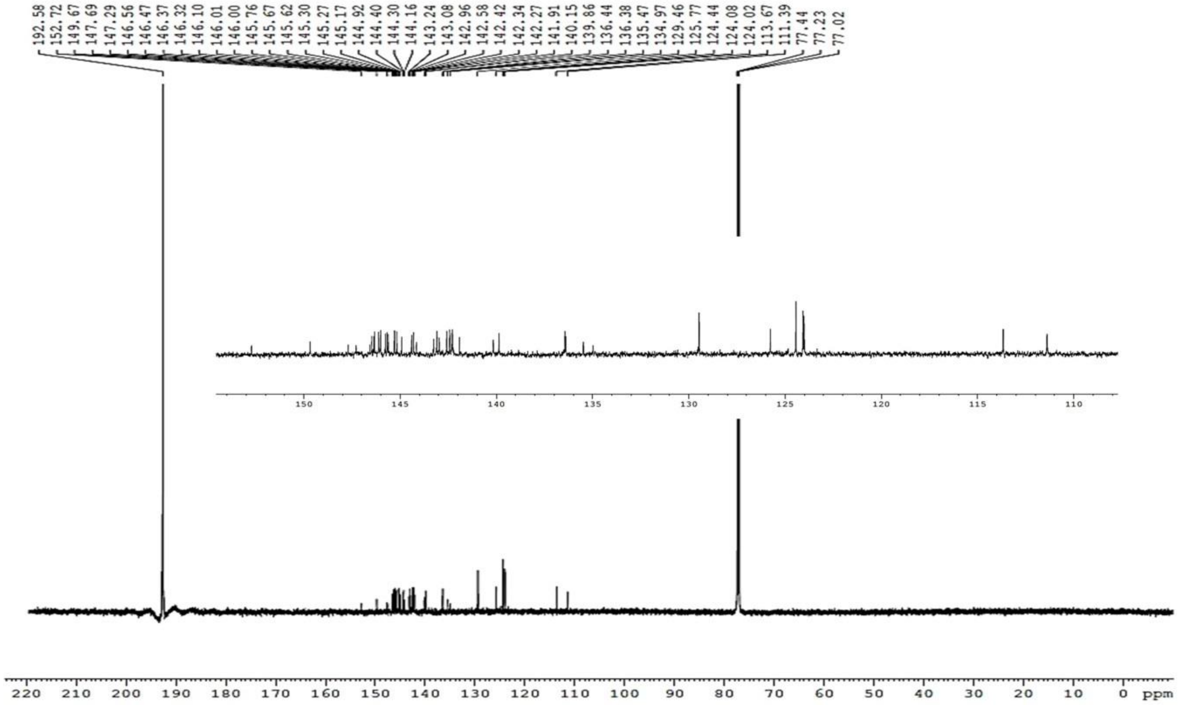

Figure 8. The ^{13}C NMR spectrum of compound **3c** (600.13 MHz, solvent CS_2 : $CDCl_3$ = 3:1)

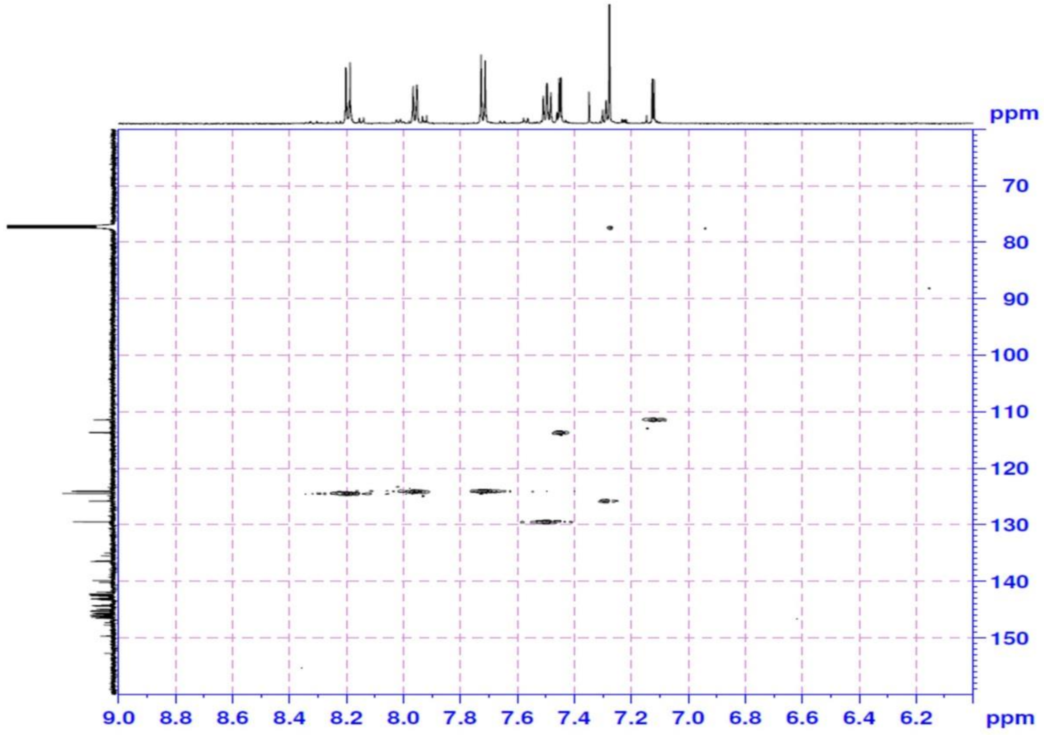

Figure 9. The HSQC spectrum of compound **3c** (600.13 MHz for ^{1}H and ^{13}C, solvent CS_2 : $CDCl_3$ = 3:1)

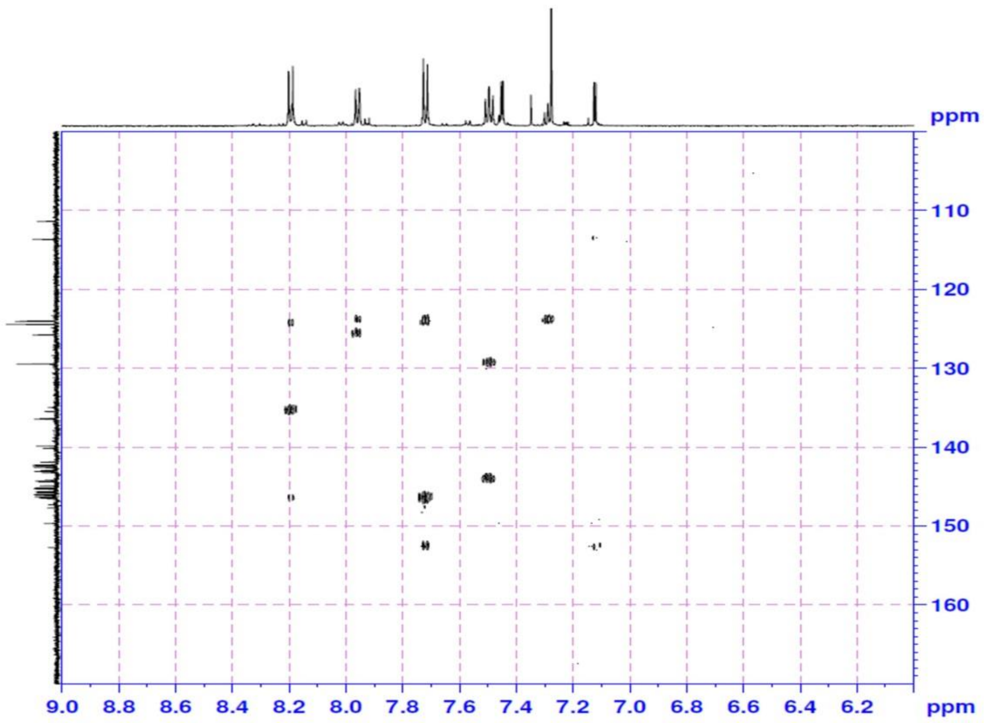

Figure 10. The HMBC spectrum of compound **3c** (600.13 MHz for ^{1}H and ^{13}C, solvent CS_2 : $CDCl_3$ = 3:1)

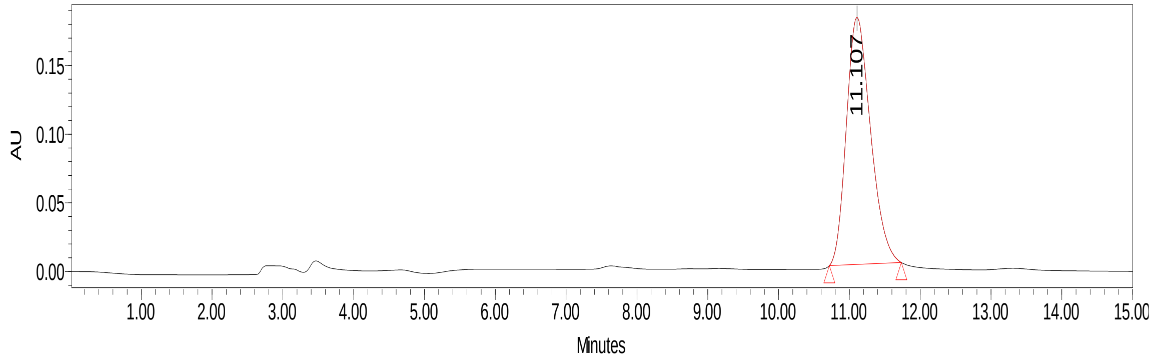

	Retention Time	Area	% Area	Height	% Height
1	11.107	4275772	100.00	180203	100.00

Figure 11. HPLC chromatogram of compound **3c**

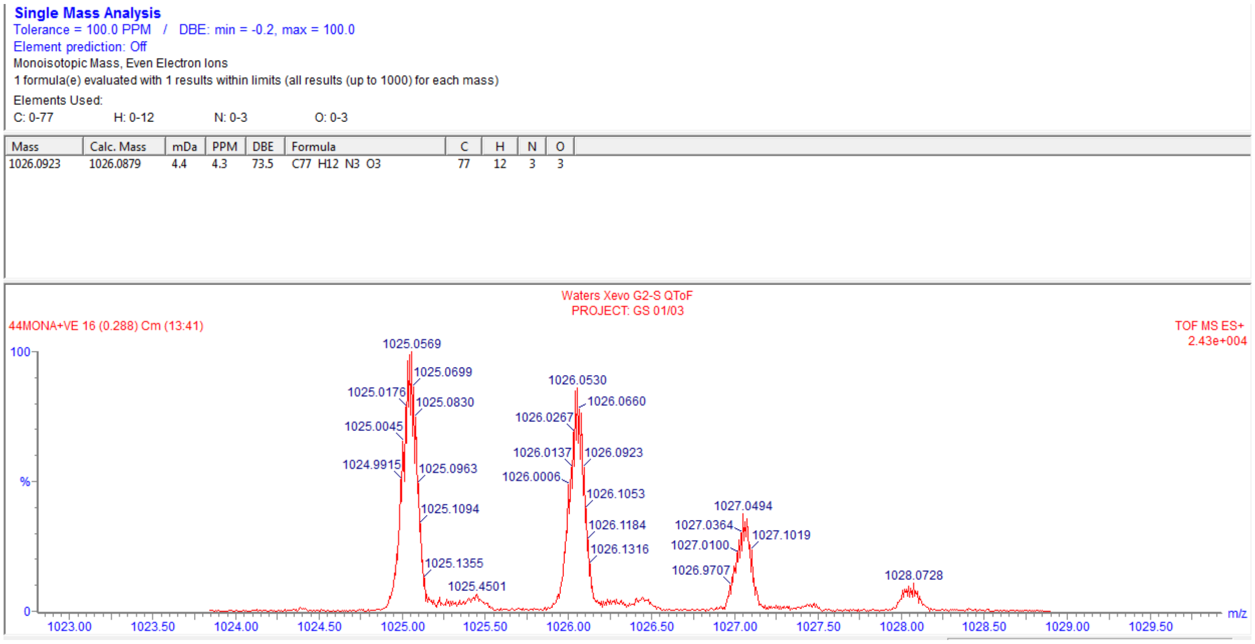

Figure 12. HRMS (ESI-TOF) of compound **3c**

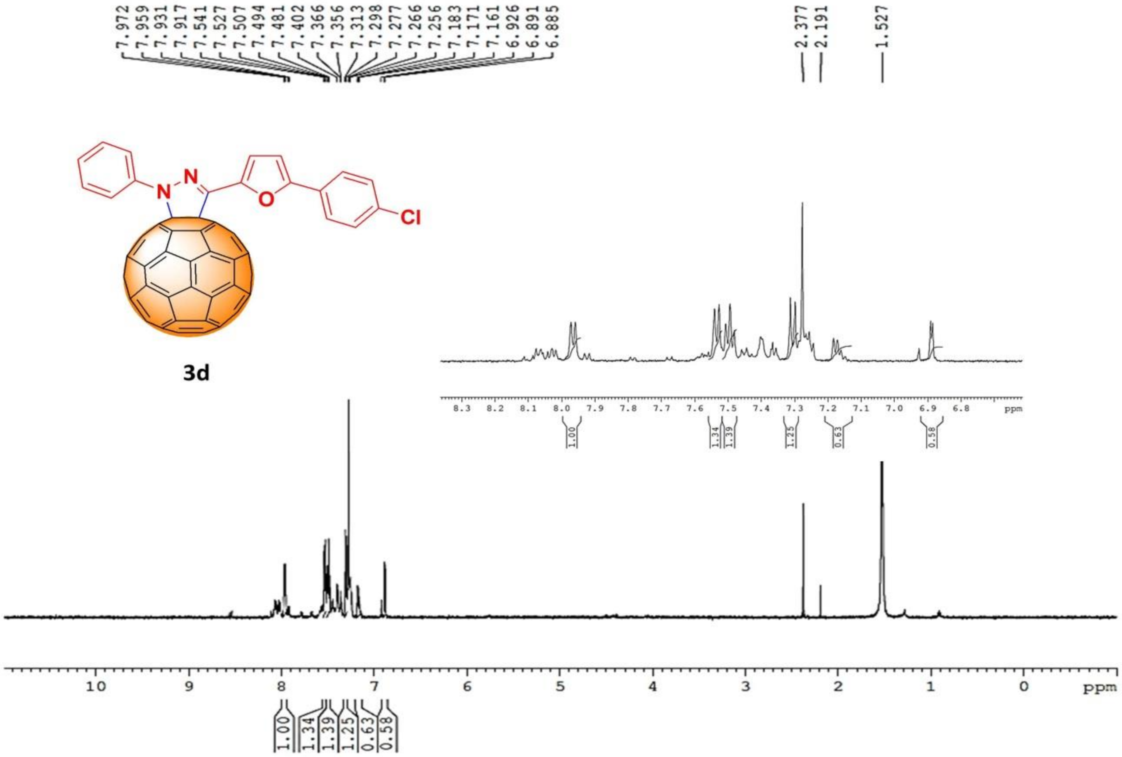

Figure 13. The ^{1}H NMR spectrum of compound **3d** (600.13 MHz, solvent $CS_2 : CDCl_3 = 3:1$)

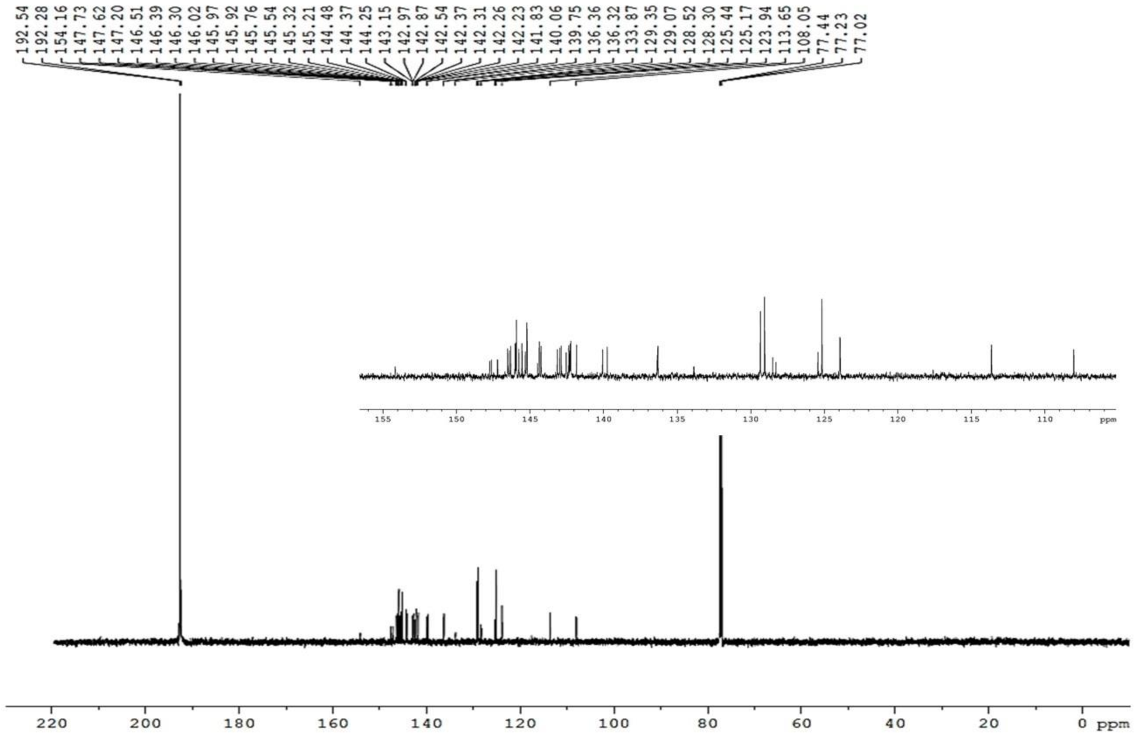

Figure 14. The ^{13}C NMR spectrum of compound **3d** (600.13 MHz, solvent $CS_2 : CDCl_3$ = 3:1)

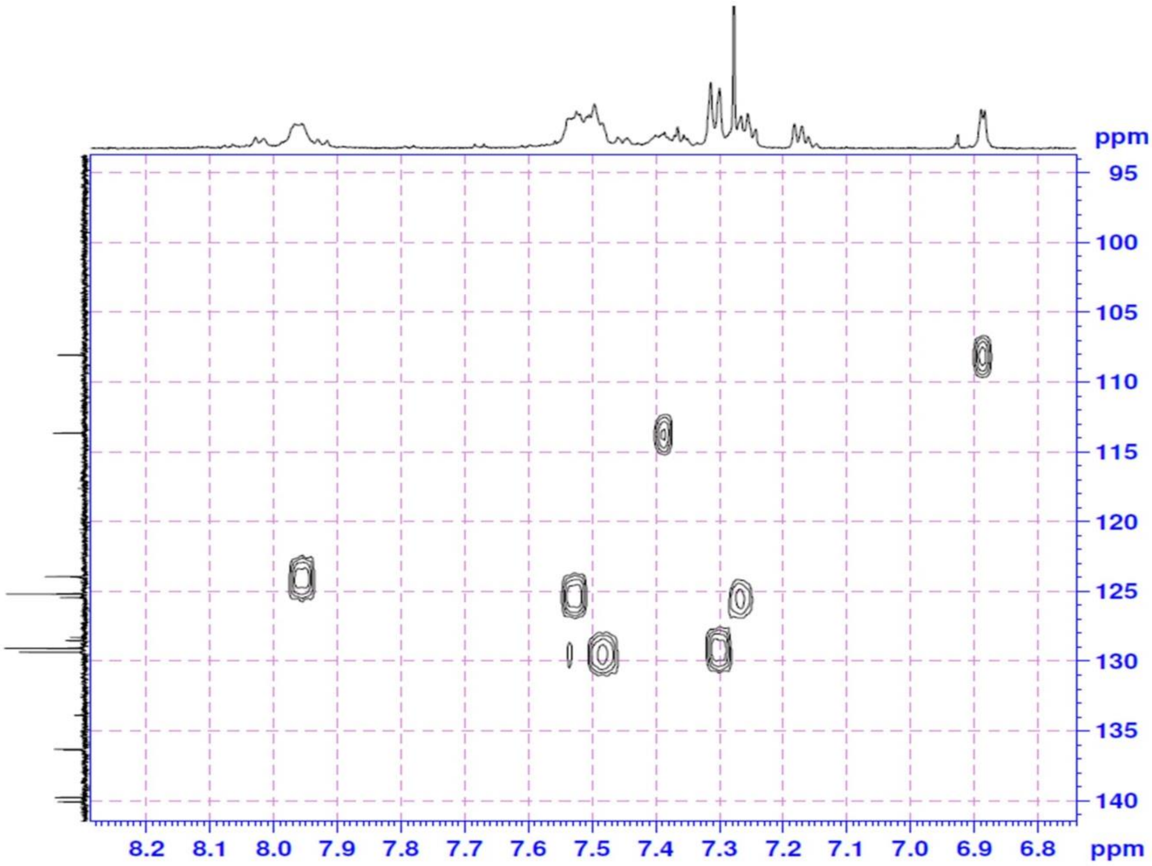

Figure 15. The HSQC spectrum of compound **3d** (600.13 MHz for ^{1}H and ^{13}C, solvent CS_2 : $CDCl_3$ = 3:1)

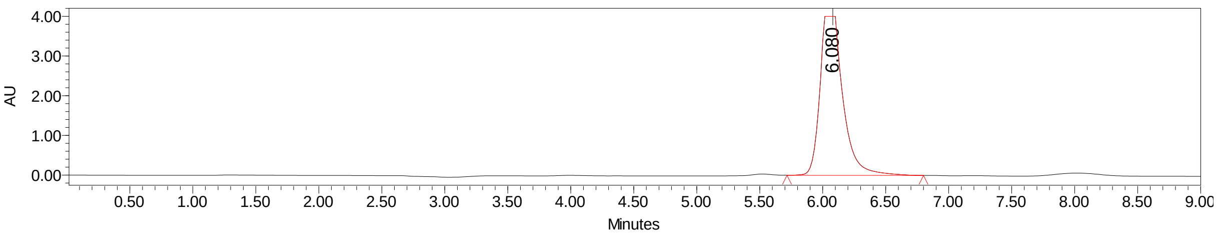

	Retention Time	Area	% Area	Height	% Height
1	6.080	51217635	100.00	4076953	100.00

Figure 16. HPLC chromatogram of compound **3d**

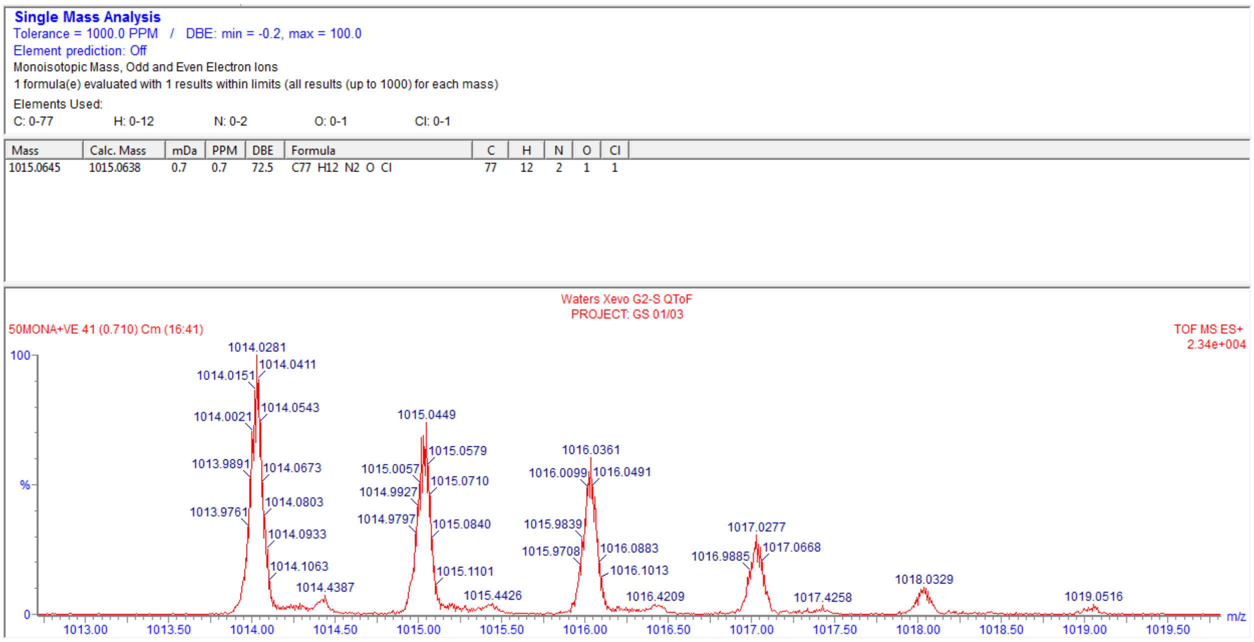

Figure 17. HRMS (ESI-TOF) of compound 3d

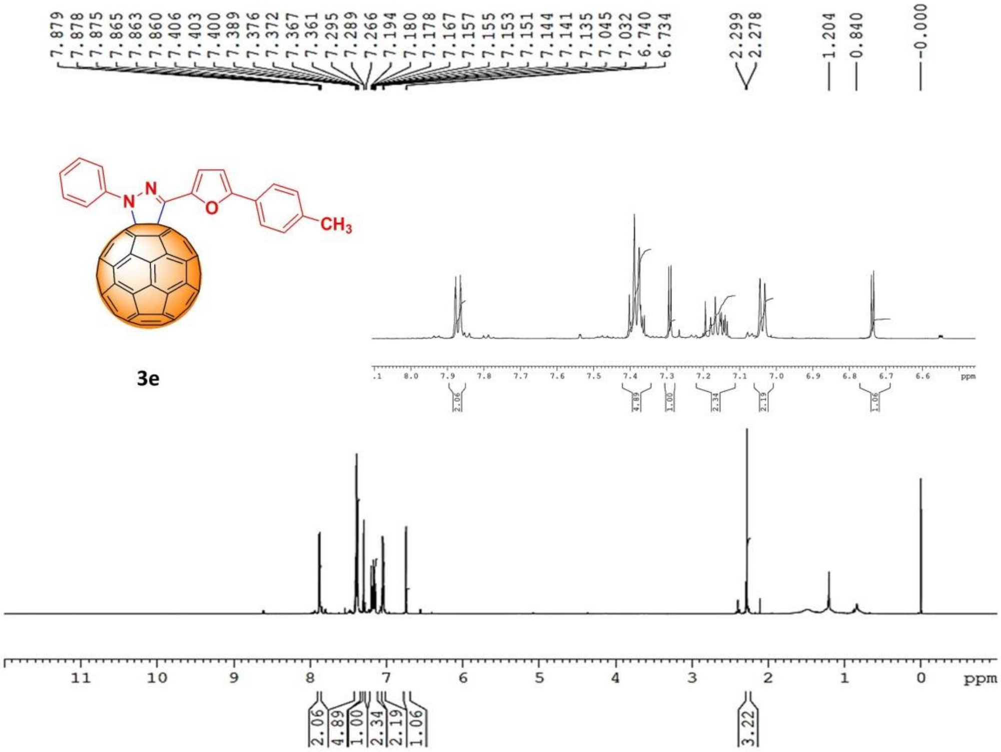

Figure 18. The ^{1}H NMR spectrum of compound **3e** (600.13 MHz, solvent CS_2 : $CDCl_3$ = 3:1)

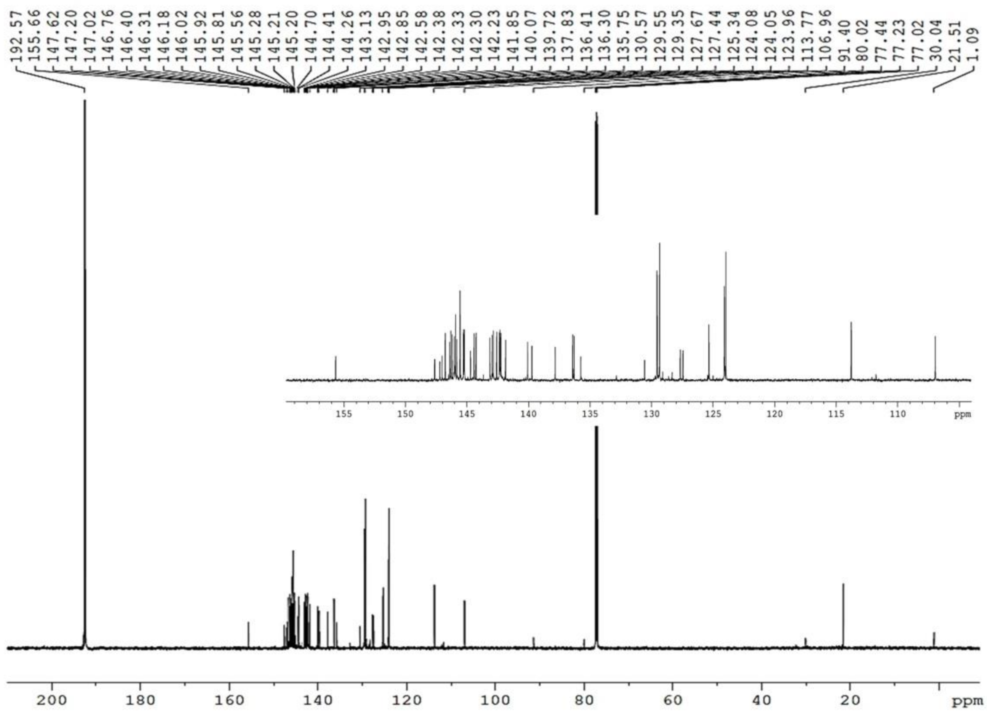

Figure 19. The ^{13}C NMR spectrum of compound **3e** (600.13 MHz, solvent CS_2 : $CDCl_3$ = 3:1)

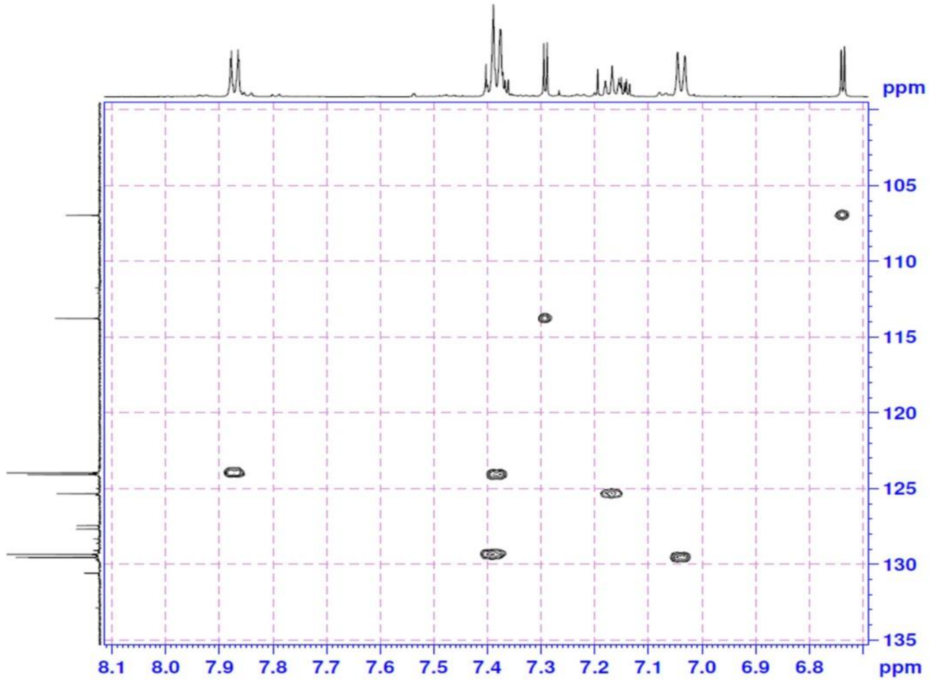

Figure 20. The HSQC spectrum of compound **3e** (600.13 MHz for ^{1}H and ^{13}C, solvent CS_2 : $CDCl_3$ = 3:1)

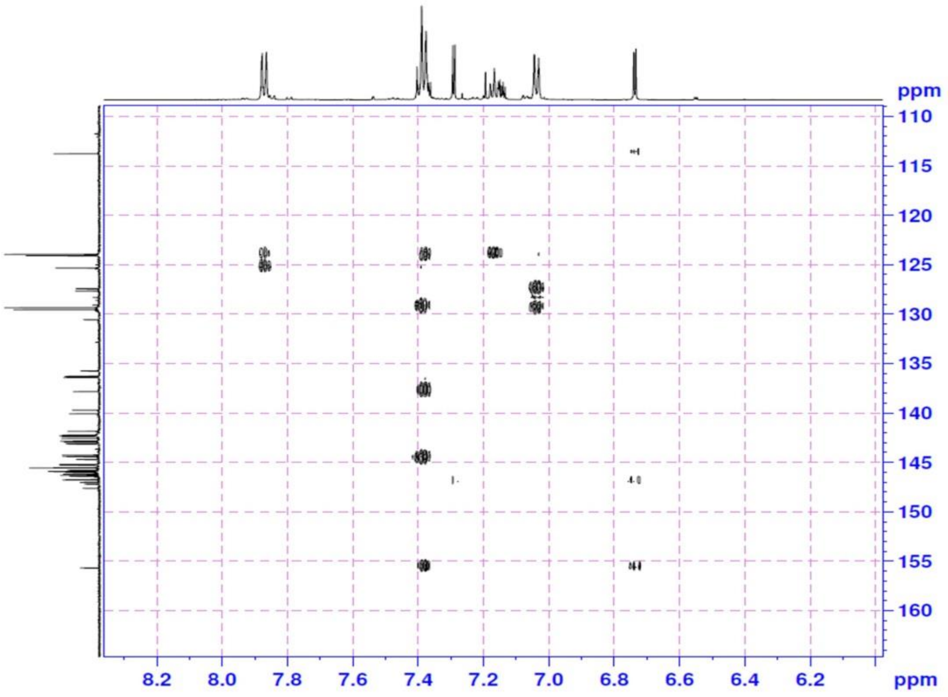

Figure 21. The HMBC spectrum of compound **3e** (600.13 MHz for ^{1}H and ^{13}C, solvent CS_2 : $CDCl_3$ = 3:1)

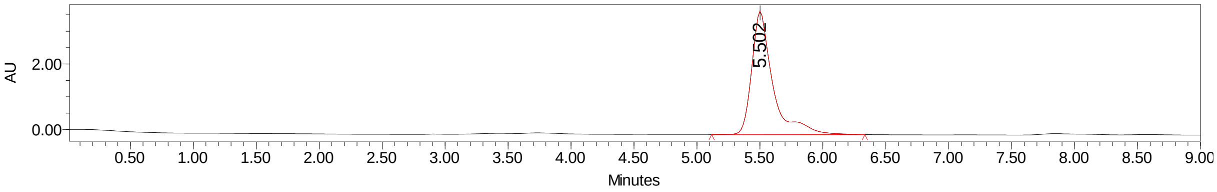

	Retention Time	Area	% Area	Height	% Height
1	5.502	43569358	100.00	3754820	100.00

Figure 22. HPLC chromatogram of compound **3e**

Single Mass Analysis
Tolerance = 100.0 PPM / DBE: min = -0.2, max = 100.0
Element prediction: Off
Monoisotopic Mass, Odd and Even Electron Ions
1 formula(e) evaluated with 1 results within limits (all results (up to 1000) for each mass)
Elements Used:
C: 0-78 H: 0-15 N: 0-2 O: 0-1

Mass	Calc. Mass	mDa	PPM	DBE	Formula	C	H	N	O
995.1231	995.1184	4.7	4.7	72.5	C78 H15 N2 O	78	15	2	1

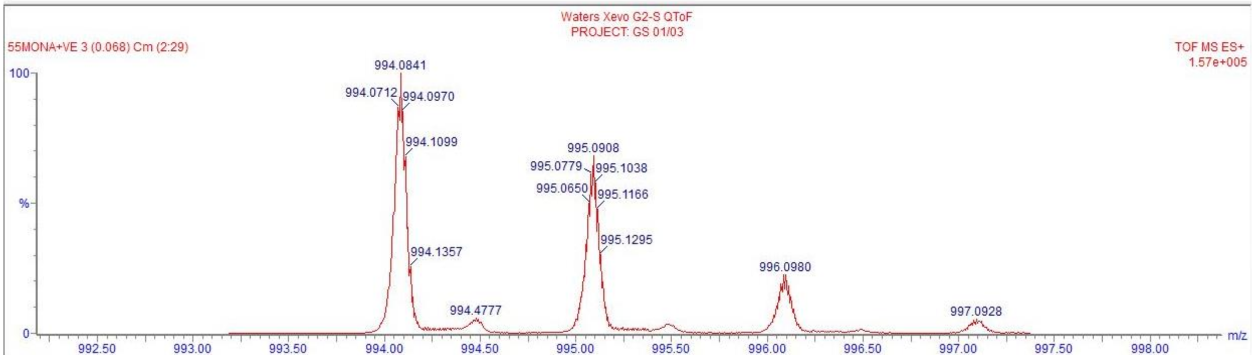

Figure 23. HRMS (ESI-TOF) of compound **3e**

Table 1. Summary on the nature and various crystallographic parameters of crystal samples of **3d** and **3e**.

**Crystal sample**	**3d**	**3e**
**Crystal data**		
Chemical formula	$C_{78}H_{12}Cl_4N_2O$	$C_{79}H_{15}Cl_3N_2O$
M_r	1134.78	1114.36
Crystal system, space group	Monoclinic, $P2_1/c$	Monoclinic, $P2_1/c$
Temperature (K)	150	150
a, *b*, *c* (Å)	15.9080 (17), 15.2055 (18), 18.4741 (19)	15.9390 (7), 15.2196 (8), 18.5972 (11)
β (°)	102.834 (7)	102.795 (7)
V (Å^3)	4357.1 (8)	4399.4 (4)
Z	4	4
Radiation type	Mo $K\alpha$	Mo $K\alpha$
μ (mm^{-1})	0.34	0.28
Crystal size (mm)	0.20 × 0.11 × 0.06	0.20 × 0.11 × 0.08
**Data collection**		
Diffractometer	Rigaku R-AXIS RAPID	Rigaku R-AXIS RAPID
Absorption correction	Multi-scan	Multi-scan
	ABSCOR (Rigaku, 1995)	*ABSCOR* (Rigaku, 1995)
T_{min}, T_{max}	0.090, 0.980	0.651, 0.978
No. of measured, independent & observed [$I > 2\sigma(I)$] reflections	26468, 7597, 3107	28310, 7579, 4260
R_{int}	0.163	0.111
$(\sin \theta/\lambda)_{max}$ (Å^{-1})	0.595	0.595
**Refinement**		
$R[F^2 > 2\sigma(F^2)]$, $wR(F^2)$, S	0.095, 0.295, 0.97	0.067, 0.166, 1.02
No. of reflections	7597	7579
No. of parameters	766	767
H-atom treatment	Constrained	Constrained
$\Delta\rho_{max}$, $\Delta\rho_{min}$ (e Å^{-3})	1.16, −0.88	0.51, −0.61

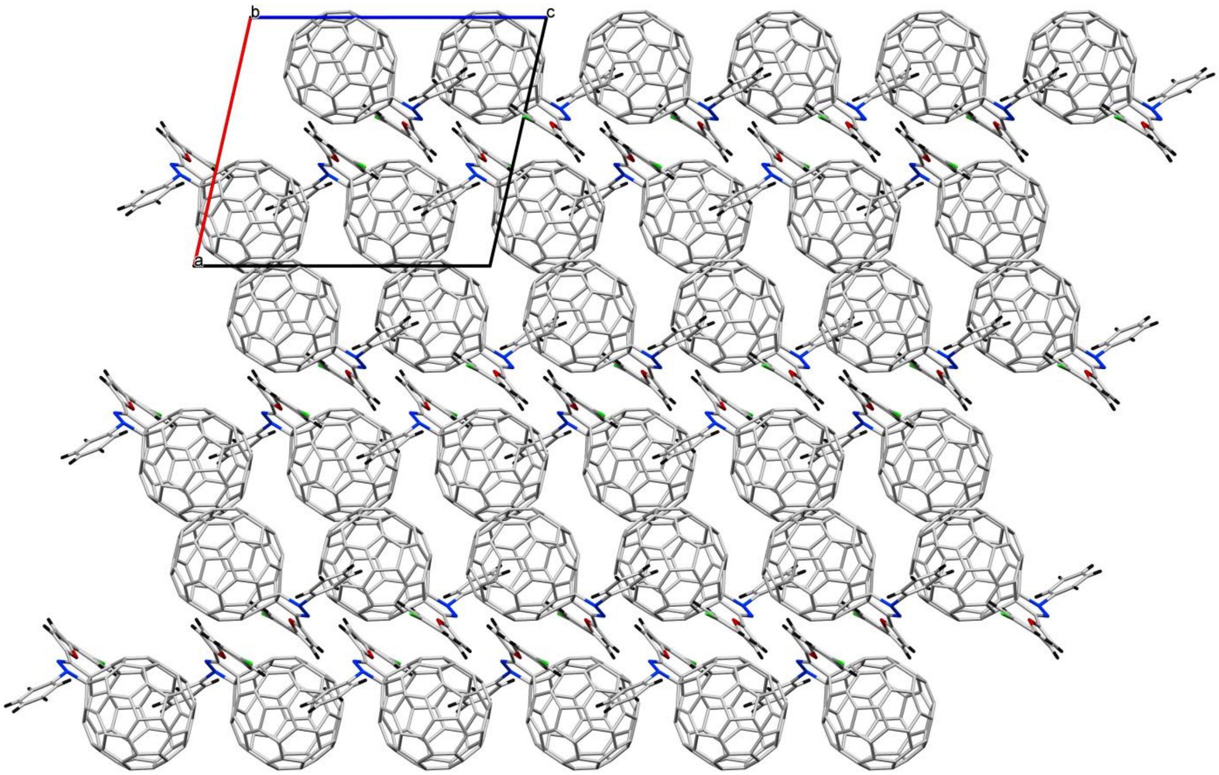

Figure 24. Packing pattern of **3d** in its crystal network

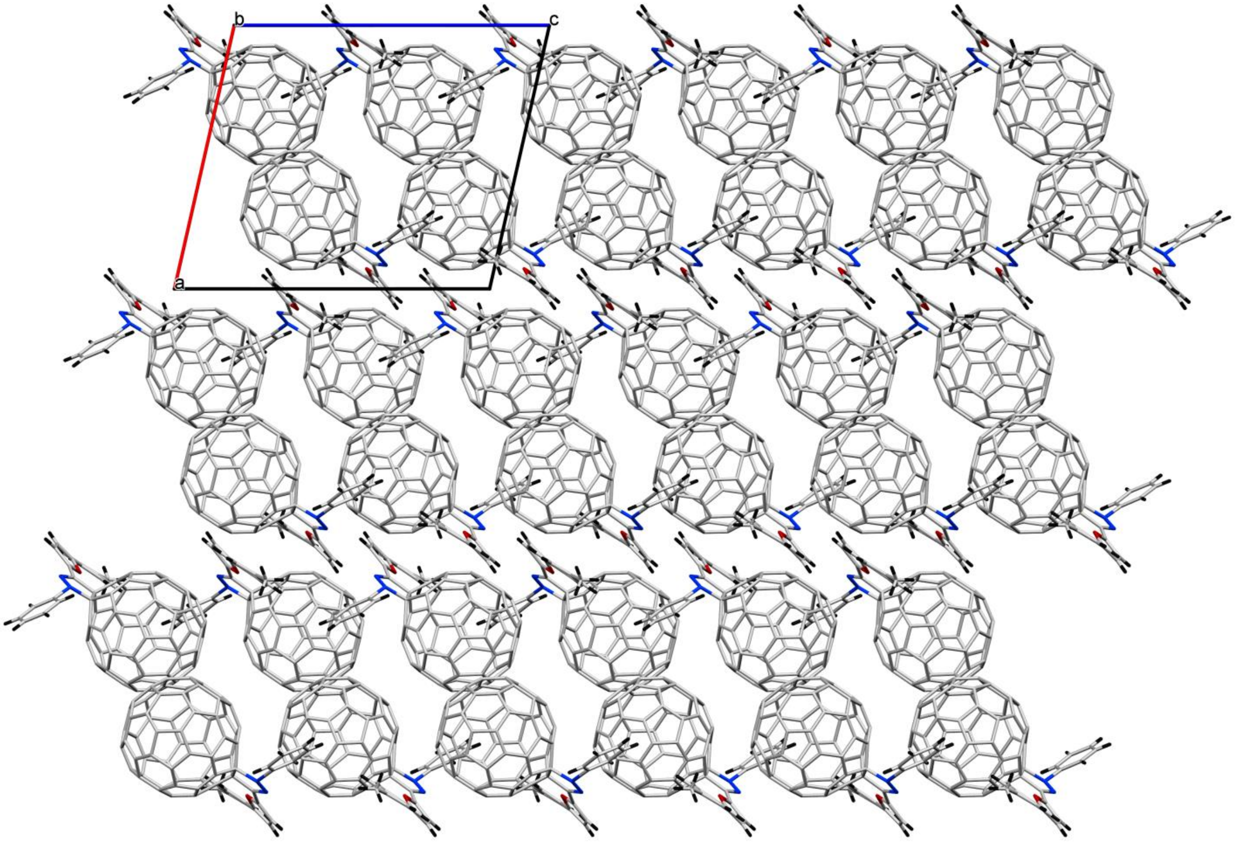

Figure 25. Packing pattern of **3e** in its crystal network.
